# Mechanical stiffness promotes skin fibrosis via Piezo1-Wnt2/Wnt11-CCL24 positive feedback loop

**DOI:** 10.1038/s41419-024-06466-3

**Published:** 2024-01-24

**Authors:** Jiahao He, Xinwei Cheng, Bin Fang, Shengzhou Shan, Qingfeng Li

**Affiliations:** Department of Plastic and Reconstructive Surgery, Shanghai Ninth People’s Hospital, Shanghai Jiao Tong University School of Medicine, 200011 Shanghai, China

**Keywords:** Mechanisms of disease, Physiology

## Abstract

Skin fibrosis is characterized by the excessive accumulation of extracellular matrix (ECM) caused by fibrotic disorders of the skin. In recent years, ECM stiffness has emerged as a prominent mechanical cue that precedes skin fibrosis and drives its progression by promoting fibroblasts activation. However, how stiffness influences fibroblasts activation for skin fibrosis progression remains unknown. Here, we report a positive feedback loop mediated by the mechanosensitive ion channel Piezo1 and aberrant tissue mechanics in driving skin fibrosis. Piezo1 is upregulated in fibrotic skin in both humans and mice. Piezo1 knockdown dermal fibroblasts lose their fibroproliferative phenotypes despite being grown on a stiffer substrate. We show that Piezo1 acts through the Wnt2/Wnt11 pathway to mechanically induce secretion of C-C motif chemokine ligand 24 (CCL24, also known as eotaxin-2), a potent cytokine associated with fibrotic disorders. Importantly, adeno-associated virus (AAV)-mediated Piezo1 knockdown ameliorated the progression of skin fibrosis and skin stiffness in mice. Overall, increased matrix stiffness promotes skin fibrosis through the inflammatory Piezo1-Wnt2/Wnt11-CCL24 pathway. In turn, a stiffer skin microenvironment increases Piezo1 expression to exacerbate skin fibrosis aggression. Therefore, targeting Piezo1 represents a strategy to break the positive feedback loop between fibroblasts mechanotransduction and aberrant tissue mechanics in skin fibrosis.

## Introduction

Skin fibrosis is a pathological process involving excessive accumulation of extracellular matrix (ECM) components caused by skin fibrotic disorders such as hypertrophic scar (HS), keloid, and scleroderma [1]. Activated fibroblasts, namely myofibroblasts, are mainly involved in the excessive production and cross-linking of ECM [2]. New insights into the pathogenesis of tissues fibrosis have been provided by the discovery that increased matrix stiffness of fibrotic tissues may contribute to a positive feedback loop with fibroblast activation [3, 4]. The accumulated deposition and remodeling of ECM continuously stimulates fibroblast activation and ECM production, further upregulating matrix stiffness, establishing this amplification loop. In this circuit, fibroblasts integrate mechanical signals from the abnormal mechanical microenvironment via mechanosensors [5]. Several key mediators, including membrane channels [6], integrins [7], focal adhesion kinase (FAK) [8] and yes-associated protein (YAP) [9], have been reported to promote fibrosis in response to matrix mechanics. However, the surface mechanosensors that directly link matrix mechanics and fibroblast activation in skin fibrosis are largely unknown.

The mechanosensitive channel Piezo1 has been shown to directly sense matrix stiffness [10]. In neural stem cell differentiation, Piezo1 provides a link between ECM mechanics and intracellular signaling [11]. In dendritic cells, Piezo1 serves as an important sensor that promotes cell metabolism and inflammatory function under environmental stiffness [12]. Monocytes also regulate angiogenesis via Piezo1 based on the mechanical properties of their surrounding matrix [13]. Importantly, Piezo1 is also essential in feedback loops that contribute to disease progression by interacting with tissue mechanics. In a Piezo1-dependent manner, glioma cells interact with aberrant glioma tissue mechanics to promote malignancy [14]. In central nervous system ageing, Piezo1 provides a negative feedback mechanism for oligodendrocyte progenitor cell proliferation and prefrontal cortex stiffness [15]. A positive feedback regulation between the cell cytoskeleton and Piezo1 activity also promotes inflammatory activation of macrophages [16]. We have previously shown that Piezo1 is involved in mechanical stretch-induced hypertrophic scar formation [17]. However, it is unknown whether aberrant matrix stiffness interacts with fibroblast Piezo1 to promote skin fibrosis. Therefore, we postulated a positive feedback loop in which Piezo1 interacts with fibrotic skin matrix stiffness to exacerbate skin fibrosis.

Here we report that Piezo1 is overexpressed in fibrotic human and mouse skin tissues, particularly in myofibroblasts. In vitro, increased substrate stiffness upregulates Piezo1 expression in human dermal fibroblasts (HDFs) and promotes HDF activation through the Piezo1 channel. Using a cytokine antibody array, we identified the Piezo1 downstream cytokine CCL24, which is linked to HDFs activation via inflammatory pathways. Furthermore, using RNA sequencing (RNA-seq), we highlight the critical role of Wnt2/Wnt11 as intracellular signaling connecting Piezo1 with CCL24 secretion. Finally, in a mouse skin fibrosis model, application of AAV-mediated Piezo1 knockdown effectively reduces skin fibrosis progression and skin stiffness. Collectively, our data provide direct insight into the molecular mechanism of skin fibrosis progression by uncovering an ECM stiffness-driven positive feedback loop that can manipulate dermal fibroblast activation by activating the Piezo1-Wnt2/Wnt11-CCL24 inflammatory signaling pathway.

## Results

### Piezo1 expression is upregulated in skin fibrosis

**Fig. 1:**
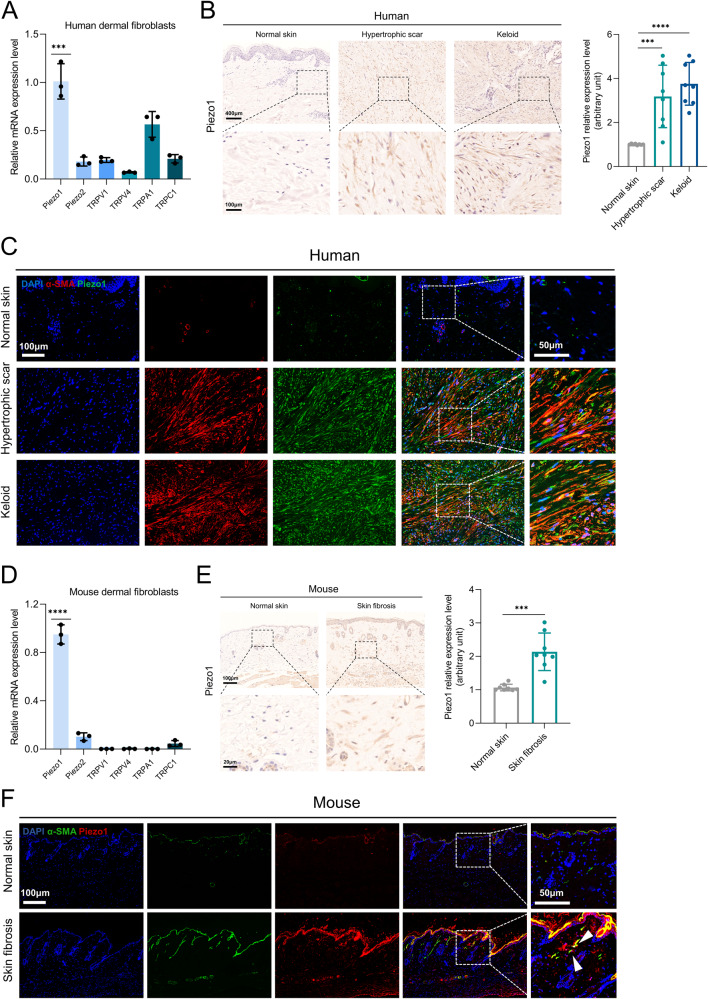
Piezo1 upregulation in skin fibrosis tissues. **A** RT–qPCR analysis of known mechanosensory ion channels from HDFs (*n* = 3). **B** Piezo1 expression in human normal skin, hypertrophic scar, keloid, and quantitative analysis. Scale bar = 400 μm, Zoom scale bar = 100 μm (*n* = 8). **C** Images of immunofluorescence co-staining of Piezo1 (green) and α-SMA (red) in human normal skin, hypertrophic scar, keloid. Scale bar = 100 μm, Zoom scale bar = 50 μm. **D** RT–qPCR analysis of known mechanosensory ion channels from MDFs (*n* = 3). **E** Piezo1 expression in mice normal skin, skin fibrosis tissues (bleomycin treatment), and quantitative analysis. Scale bar = 100 μm, Zoom scale bar = 20 μm (*n* = 8). **F** Images of immunofluorescence co-staining of Piezo1 (green) and α-SMA (red) in mice normal skin, skin fibrosis tissues (bleomycin treatment). Scale bar = 100 μm, Zoom scale bar = 50 μm. The results are expressed as the means with SD. “*n*” meaning (PCR and immunostaining: Statistics were collected with “*n*” independent biological samples). Two-tailed t-test or One-way ANOVA is used for all analyses. ***P < 0.005, ****P < 0.001.

To investigate mechanosensation in HDFs, we first examined the expression of mechanically activated cation channels (MACs) in HDFs. RT-qPCR confirmed that Piezo1 expression is higher than other reported MACs (Fig. 1A). We then investigated the protein level of Piezo1 in normal skin, hypertrophic scar and keloid tissues from humans. Western blot confirmed that Piezo1 is overexpressed in human fibrotic skin tissues (Supplementary Fig. 1A). Immunohistochemical analysis confirmed an increase in Piezo1 in human fibrotic skin tissues (Fig. 1B). As shown by α-SMA/Piezo1 immunofluorescence co-staining, we observed that Piezo1 is highly expressed in myofibroblasts in human fibrotic skin tissues (Fig. 1C). We also examined Piezo1 expression in MDFs. MDFs also show high expression of Piezo1 and negligible levels of other MACs (Fig. 1D). Western blot confirmed that Piezo1 is overexpressed in mouse fibrotic skin tissues (Supplementary Fig. 1B). Immunohistochemical analysis revealed that Piezo1 is overexpressed throughout the dermis in the bleomycin-induced skin fibrosis mouse model (Fig. 1E). Immunofluorescence staining with Piezo1 and α-SMA shows that Piezo1 is overexpressed in mouse dermal myofibroblasts, similar to the phenomenon observed in human fibrotic skin tissue (Fig. 1F). Taken together, these results suggest that Piezo1 may be associated with mechanical stiffness-mediated progression of skin fibrosis.

### Increased matrix stiffness stimulates Piezo1 overexpression and Piezo1-dependent HDFs activation

**Fig. 2:**
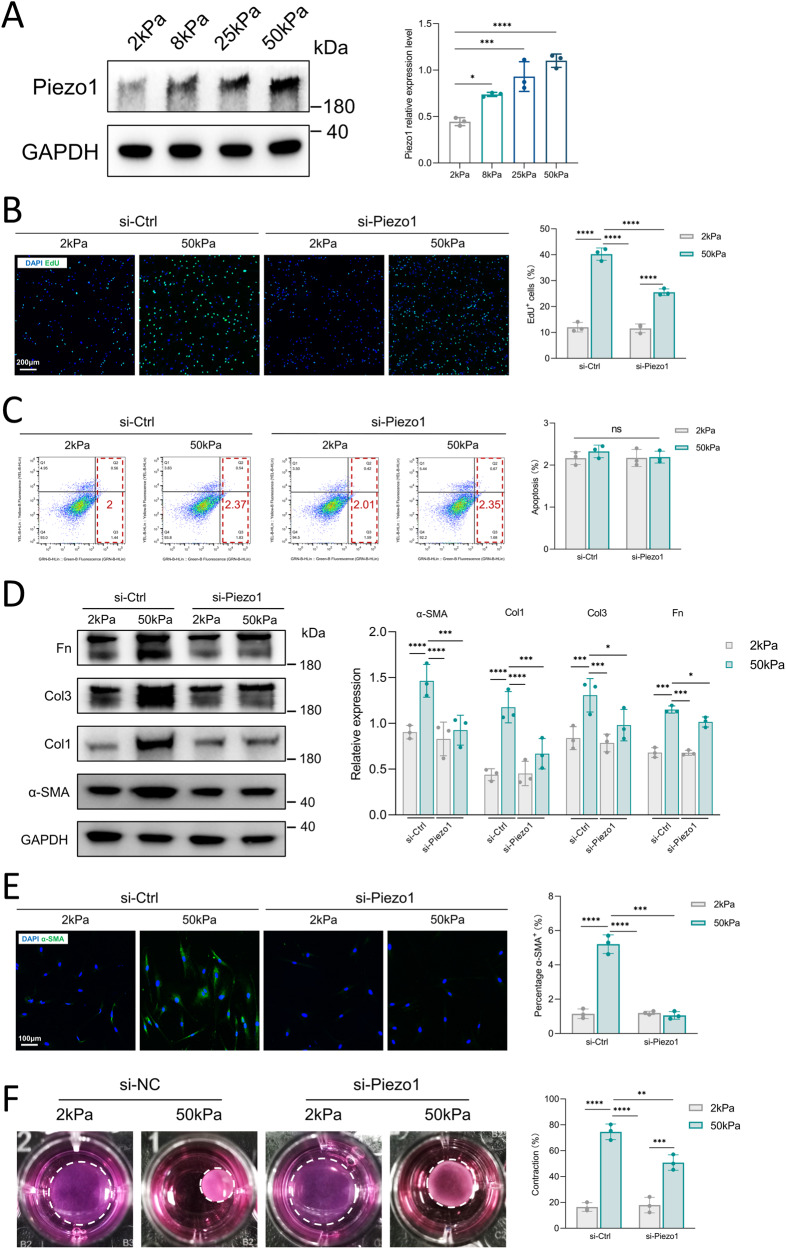
The influence of increased matrix stiffness on Piezo1 expression and Piezo1-mediated HDFs activation. **A** Effect of increased matrix stiffness on Piezo1 protein expression as demonstrated by western blotting and quantitative analysis (*n* = 3). **B** HDFs proliferation was detected by EdU immunofluorescent staining and quantitative analysis of the percentage of EdU-positive cells. Scale bar: 200 μm (*n* = 3). **C** Apoptosis was detected by Annexin V and flow cytometry (*n* = 3). **D** Expression of GAPDH, α-SMA, Col1, Col3, and Fn was detected by western blotting and quantitative analysis of protein expression (*n* = 3). **E** Immunofluorescent staining for α-SMA and quantitative analysis of the percentage of α-SMA positive cells. Scale bar: 100 μm (*n* = 3). **F** Images and quantitative analysis of fibroblast contraction in three-dimensional collagen lattices (*n* = 3). The results are expressed as the means with SD. “*n*” meaning (Western blot: Statistics were collected with “*n*” independent gel blots; PCR, Flow cytometry, immunostaining, and photograph: Statistics were collected with “*n*” independent biological samples). One-way ANOVA is used for all analyses. *P < 0. 05, **P < 0.01, ***P < 0.005, ****P < 0.001.

Previous reports have described that increased matrix stiffness leads to Piezo1 overexpression [18]. Therefore, we speculate that aberrant matrix stiffness of fibrotic skin promotes Piezo1 expression in vivo. We then cultured HDFs on hydrogels of varying stiffness from 2 to 50 kPa (mimicking the elastic range of normal skin and fibrotic skin tissue). Notably, we observed that Piezo1 expression was upregulated by the increased matrix stiffness (Fig. 2A). We then investigated the role of Piezo1 in the activation of HDFs induced by increased matrix stiffness. The EdU proliferation assay showed that increased substrate stiffness-induced cell proliferation was downregulated after si-Piezo1 treatment (Fig. 2B). However, apoptosis of HDFs showed no significant difference in all groups (Fig. 2C). Increased matrix stiffness stimulated the expression of ECM components (fibronectin, Col3 and Col1) and α-SMA in HDFs, and Piezo1 knockdown abolished the overexpression of these proteins (Fig. 2D). Similarly, α-SMA immunostaining and collagen contraction assay confirmed that increased matrix stiffness could upregulate α-SMA expression through the Piezo1 channel (Fig. 2E, F). Taken together, these results suggest that mechanical stiffness-induced activation of HDFs could be regulated by Piezo1 activity.

### CCL24 was identified as a Piezo1 downstream cytokine in response to mechanical stiffness

**Fig. 3:**
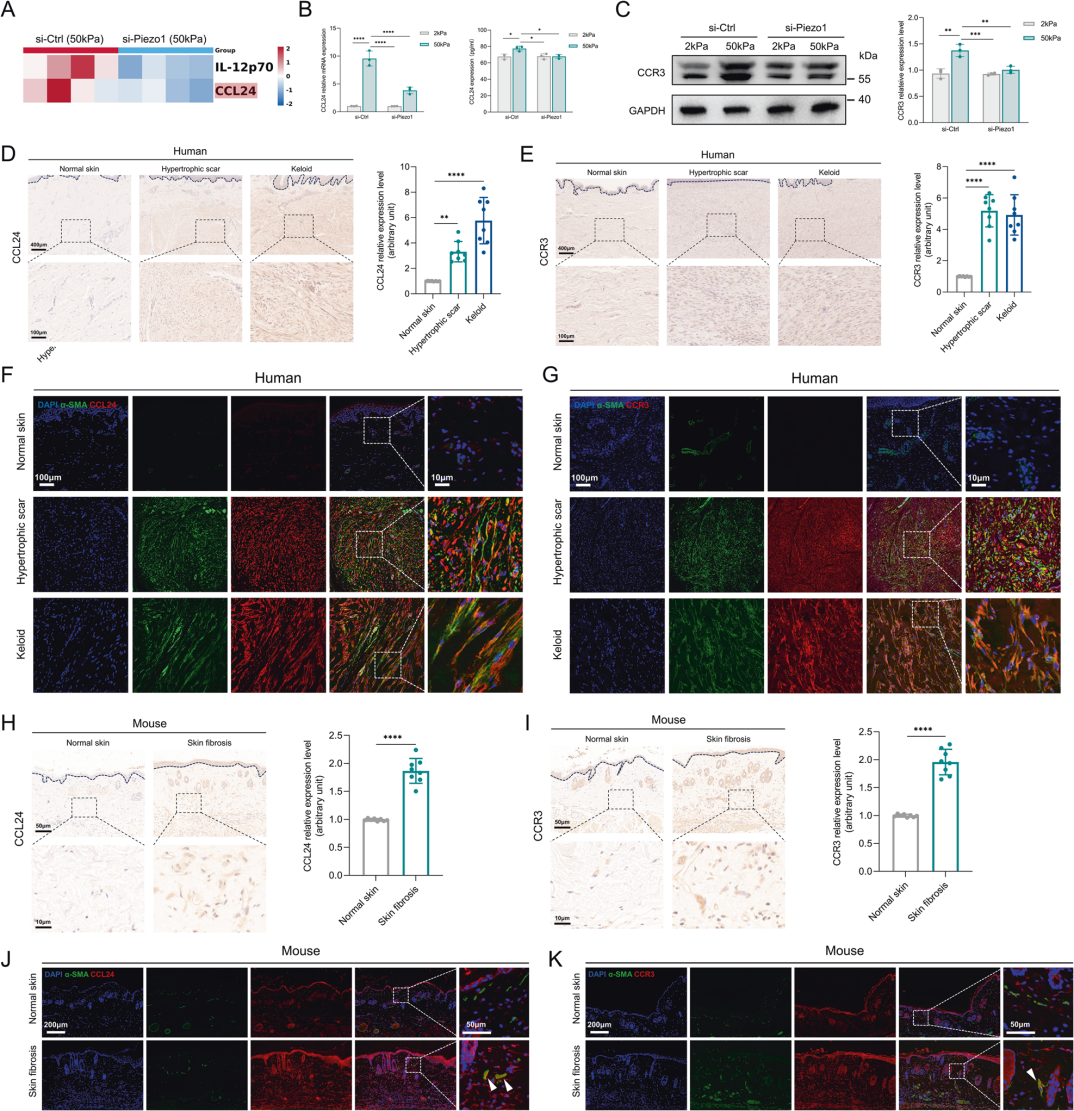
CCL24 was identified as a Piezo1 downstream inflammatory cytokines. **A** Human inflammatory cytokines were differentially secreted in the supernatant of cultured si-Ctrl HDFs vs si-Piezo1 HDFs grown on 50 kPa (*n* = 3). **B** CCL24 mRNA expression was detected by RT-PCR and CCL24 concentration in the medium was detected by ELISA (*n* = 3). **C** Expression of CCR3 was detected by western blotting and quantitative analysis of protein expression (*n* = 3). **D** CCL24 expression in human normal skin, hypertrophic scar, keloid, and quantitative analysis. Scale bar = 400 μm, Zoom scale bar = 100 μm (*n* = 8). **E** CCR3 expression in human normal skin, hypertrophic scar, keloid, and quantitative analysis. Scale bar = 400 μm, Zoom scale bar = 100 μm (*n* = 8). **F** Images of immunofluorescence co-staining of CCL24 (red) and α-SMA (green) in human normal skin, hypertrophic scar, keloid. Scale bar = 100 μm, Zoom scale bar = 10 μm. **G** Images of immunofluorescence co-staining of CCR3 (red) and α-SMA (green) in human normal skin, hypertrophic scar, keloid. Scale bar = 100 μm, Zoom scale bar = 10 μm. **H** CCL24 expression in mice normal skin, skin fibrosis tissues (bleomycin treatment) and quantitative analysis. Scale bar = 50 μm, Zoom scale bar = 10 μm (*n* = 8). **I** CCR3 expression in mice normal skin, skin fibrosis tissues (bleomycin treatment) and quantitative analysis. Scale bar = 50 μm, Zoom scale bar = 10 μm (*n* = 8). **J** Images of immunofluorescence co-staining of CCL24 (red) and α-SMA (green) in mice normal skin, skin fibrosis tissues (bleomycin treatment). Scale bar = 200 μm, Zoom scale bar = 50 μm. **K** Images of immunofluorescence co-staining of CCR3 (red) and α-SMA (green) in mice normal skin, skin fibrosis tissues (bleomycin treatment). Scale bar = 200 μm, Zoom scale bar = 50 μm. The results are expressed as the means with SD. “*n*” meaning (Western blot: Statistics were collected with “*n*” independent gel blots; PCR, inflammatory cytokines panel, ELISA, and immunostaining: Statistics were collected with “*n*” independent biological samples). Two-tailed t-test or One-way ANOVA is used for all analyses. *P < 0.05, **P < 0.01, ***P < 0.005, ****P < 0.001.

As inflammatory mechanisms are strongly implicated in the progression of skin fibrosis [19], we investigated whether Piezo1 modulates cytokine signaling. Notably, as suggested by our cytokine antibody arrays, levels of CCL24 (also known as eotaxin-2), a chemokine strongly associated with dermal and pulmonary fibrosis [20], were lower in Piezo1 knockdown HDFs grown on higher substrate stiffness (Fig. 3A). Consistent with the cytokine antibody arrays, PCR and in vitro ELISA assay further confirmed that increased matrix stiffness could stimulate CCL24 secretion through Piezo1 activation (Fig. 3B). In addition, expression of CCR3 (the surface receptor for CCL24) was reduced in Piezo1-deficient HDFs cultured on higher mechanical stiffness (Fig. 3C). Importantly, CCL24/CCR3 expression is present in fibrotic human and mouse skin tissues, particularly in myofibroblasts (Fig. 3D–K). To fully elucidate whether CCL24 could directly modulate HDFs activation, human CCL24 protein was added to the HDFs culture media. We observed that CCL24 promoted HDFs proliferation (Supplementary Fig. 2A, B), ECM components/α-SMA production (Supplementary Fig. 2C–F) and cellular contraction (Supplementary Fig. 2G, H) in a dose- and time-dependent manner. Collectively, these results indicate that CCL24-dependent inflammatory pathways play a critical role in Piezo1-mediated HDFs activation in response to mechanical stiffness.

### Wnt2/Wnt11 pathways were identified as a Piezo1 downstream pathway in response to mechanical stiffness

**Fig. 4:**
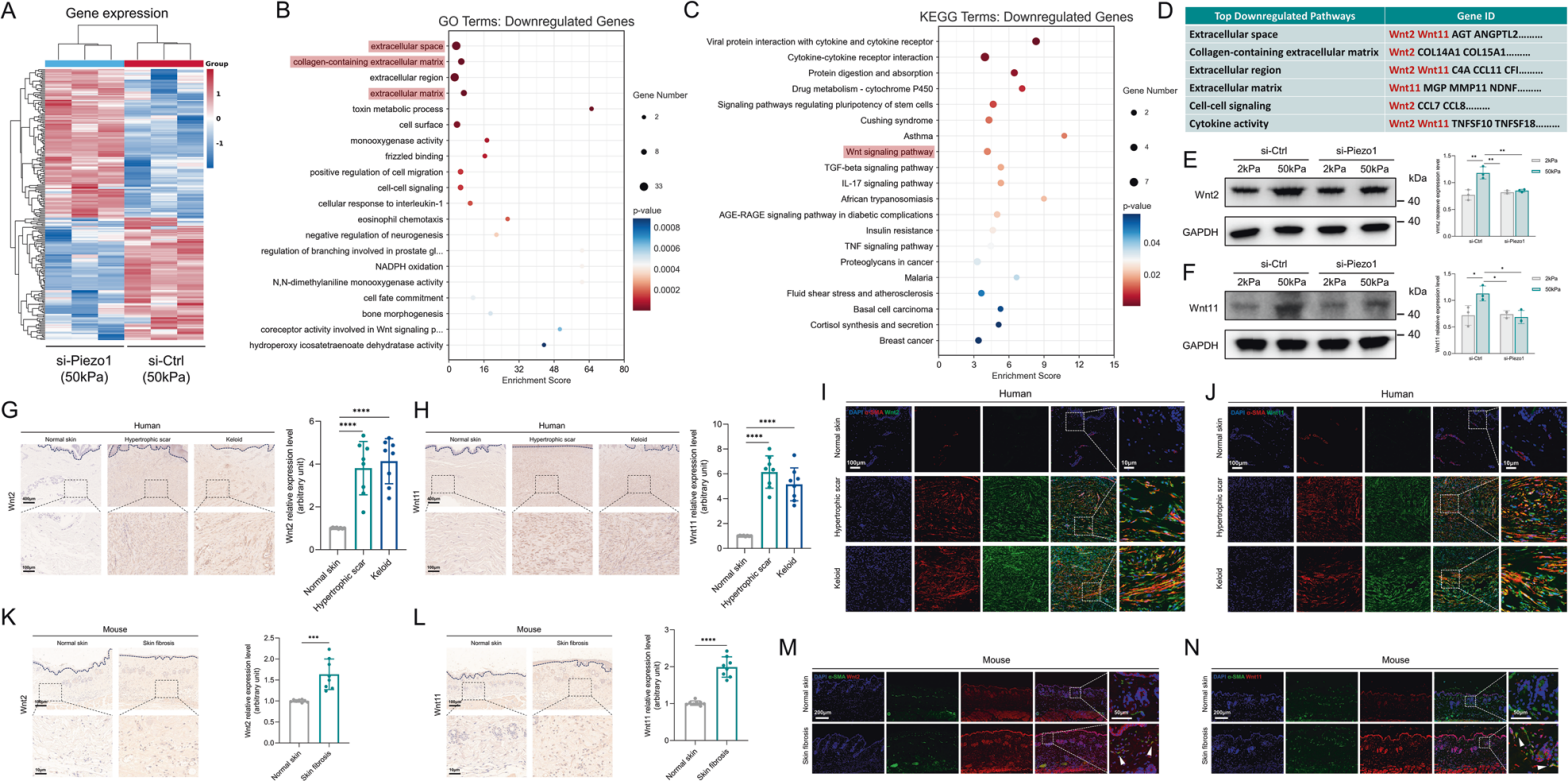
Piezo1-mediated Wnt2/Wnt11 mechanoresponsive pathways in HDFs. **A** Heatmap showing hierarchical clustering of differentially expressed genes (fold change > 2) between si-Piezo1 HDFs vs si-Ctrl HDFs grown on 50 kPa (*n* = 3). **B** GO enrichment analysis showing the most downregulated pathways (based on RNA-seq data) between si-Piezo1 HDFs vs si-Ctrl HDFs grown on 50 kPa. **C** KEGG enrichment analysis showing the most downregulated pathways (based on RNA-seq data) between si-Piezo1 HDFs vs si-Ctrl HDFs grown on 50 kPa. **D** Wnt2/Wnt11 expression in the most downregulated pathways of GO enrichment analysis. **E** Expression of Wnt2 was detected by western blotting and quantitative analysis of protein expression (*n* = 3). **F** Expression of Wnt11 was detected by western blotting and quantitative analysis of protein expression (*n* = 3). **G** Wnt2 expression in human normal skin, hypertrophic scar, keloid, and quantitative analysis. Scale bar = 400 μm, Zoom scale bar = 100 μm (*n* = 8). **H** Wnt11 expression in human normal skin, hypertrophic scar, keloid, and quantitative analysis. Scale bar = 400 μm, Zoom scale bar = 100 μm (*n* = 8). **I** Images of immunofluorescence co-staining of Wnt2 (green) and α-SMA (red) in human normal skin, hypertrophic scar, keloid. Scale bar = 100 μm, Zoom scale bar = 10 μm. **J** Images of immunofluorescence co-staining of Wnt11 (green) and α-SMA (red) in human normal skin, hypertrophic scar, keloid. Scale bar = 100 μm, Zoom scale bar = 10 μm. **K** Wnt2 expression in mice normal skin, skin fibrosis tissues (bleomycin treatment) and quantitative analysis. Scale bar = 50 μm, Zoom scale bar = 10 μm (*n* = 8). **L** Wnt11 expression in mice normal skin, skin fibrosis tissues (bleomycin treatment), and quantitative analysis. Scale bar = 50 μm, Zoom scale bar = 10 μm (*n* = 8). **M** Images of immunofluorescence co-staining of Wnt2 (red) and α-SMA (green) in mice normal skin, skin fibrosis tissues (bleomycin treatment). Scale bar = 200 μm, Zoom scale bar = 50 μm. **N** Images of immunofluorescence co-staining of Wnt11 (red) and α-SMA (green) in mice normal skin, skin fibrosis tissues (bleomycin treatment). Scale bar = 200 μm, Zoom scale bar = 50 μm. The results are expressed as the means with SD. “*n*” meaning (Western blot: Statistics were collected with “*n*” independent gel blots; RNA-seq and immunostaining: Statistics were collected with “*n*” independent biological samples). Two-tailed *t* test or One-way ANOVA is used for all analyses. *P < 0.05, **P < 0.01, ***P < 0.005, ****P < 0.001.

To elucidate candidate pathways linking Piezo1 to CCL24 secretion, we performed RNA-seq between si-Piezo1 HDFs and si-Ctrl HDFs grown on higher substrate stiffness (Fig. 4A). As expected, in GO enrichment analysis, Piezo1 knockdown mainly decreased ECM production, consistent with our in vitro assay (Fig. 4B). In KEGG enrichment analysis, Piezo1 knockdown resulted in downregulation of cytokine-cytokine receptor interaction, suggesting a role for Piezo1 in cytokine-based fibrosis paradigms (Fig. 4C). We further focused on the downregulation of “Wnt signaling pathway” in the KEGG enrichment analysis, as Wnt signaling pathway was identified as a mechanoresponsive pathway [21] and a downstream mediator of Piezo1 according to previous report [22]. In the Wnt pathway, Wnt2 and Wnt11 were highly expressed. Importantly, Wnt2 and Wnt11 were also highly expressed in GO enrichment top-down regulated pathways such as “extracellular space”, “collagen-containing extracellular matrix”, “extracellular region”, “extracellular matrix”, “cell–cell signaling” and “cytokine activity”, which are related to ECM synthesis and cytokine signaling (Fig. 4D). Thus, Wnt2/Wnt11 are key mediators in Piezo1-induced ECM production and inflammatory mechanism. Western blotting confirmed that mechanical stiffness upregulates Wnt2/Wnt11 expression through Piezo1 (Fig. 4E, F). In human and mouse fibrotic skin tissues, Wnt2/Wnt11 are also overexpressed in the dermis and myofibroblasts (Fig. 4G–N). Thus, these results demonstrate that Wnt2/Wnt11 serve as a key target of Piezo1 mechanotransduction.

### The role of inflammatory Piezo1–Wnt2/Wnt11–CCL24 pathways in HDFs activation

**Fig. 5:**
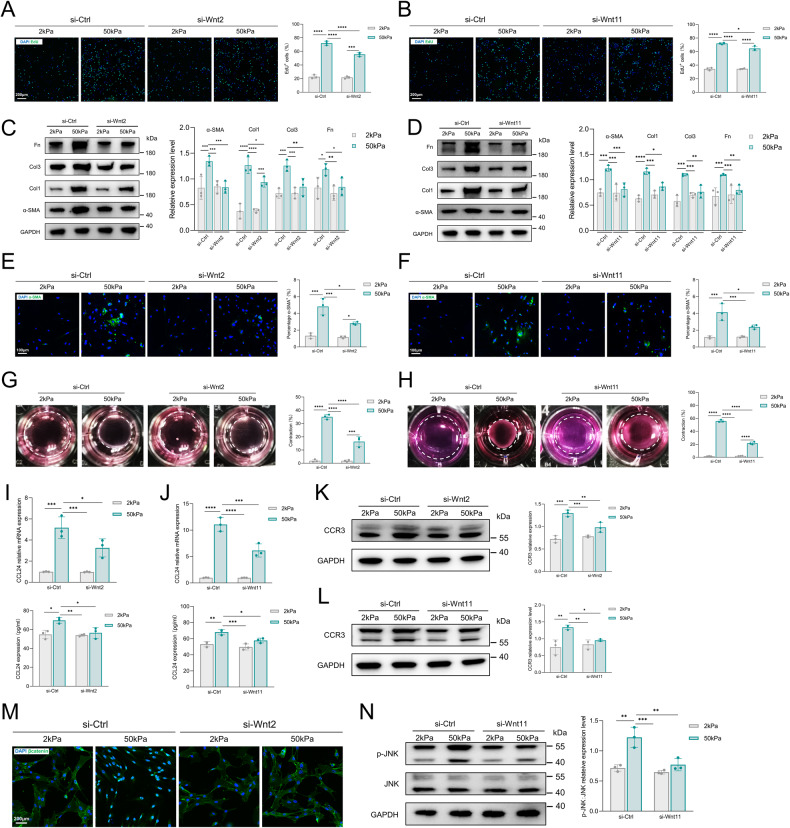
Wnt2/Wnt11 mechanoresponsive pathways in HDFs activation. **A**, **B** HDFs proliferation was detected by EdU immunofluorescent staining and quantitative analysis of the percentage of EdU-positive cells. Scale bar: 200 μm (*n* = 3). **C**, **D** Expression of GAPDH, α-SMA, Col1, Col3, and Fn was detected by western blotting and quantitative analysis of protein expression (*n* = 3). **E**, **F** Immunofluorescent staining for α-SMA and quantitative analysis of the percentage of α-SMA positive cells. Scale bar: 100 μm (*n* = 3). **G**, **H** Images and quantitative analysis of fibroblast contraction in three-dimensional collagen lattices (*n* = 3). **I**, **J** CCL24 mRNA expression was detected by RT-PCR and CCL24 concentration in the medium was detected by ELISA (*n* = 3). **K**, **L** Expression of CCR3 was detected by western blotting and quantitative analysis of protein expression (*n* = 3). **M** Immunofluorescent staining for β-catenin in HDFs. Scale bar: 200 μm. **N** Expression of GAPDH, JNK, and p-JNK was detected by western blotting and quantitative analysis of protein expression (*n* = 3). The results are expressed as the means with SD. One-way ANOVA is used for all analyses. “*n*” meaning (Western blot: Statistics were collected with “*n*” independent gel blots; PCR, ELISA, immunostaining, and photograph: Statistics were collected with “*n*” independent biological samples). *P < 0. 05, **P < 0.01, ***P < 0.005, ****P < 0.001.

We next investigated the role of Wnt2/Wnt11 in mechanical stiffness-induced HDFs activation. EdU staining revealed that increased substrate stiffness-induced cell proliferation was downregulated after si-Wnt2 or si-Wnt11 treatment (Fig. 5A, B). Wnt2/Wnt11 is also involved in mechanical stiffness-induced activation of HDFs, including synthesis of ECM components, α-SMA production and contractility (Fig. 5C–H). Most importantly, Wnt2/Wnt11 inhibition blocked mechanical stiffness-induced CCL24 release (Fig. 5I, J) and CCR3 overexpression (Fig. 5K, L), highlighting the key role of the Piezo1-Wnt2/Wnt11-CCL24 axis in mechanotransduction and inflammation in HDFs. Wnt signaling has been reported to include the canonical β-catenin pathway and non-canonical pathways. In fibroblast activation, Wnt2 is usually involved in the canonical β-catenin pathway and Wnt11 is usually involved in the non-canonical pathways (activation of JUN N-terminal kinase (JNK) via phosphorylation). In our data, we confirmed that mechanical stiffness could stimulate the Wnt2/β-catenin pathway (Fig. 5M) and the Wnt11/JNK pathway (Fig. 5N). Taken together, these data highlight the critical role of the Piezo1-Wnt2/Wnt11-CCL24 pathway in the activation of HDFs.

### Overexpressed Wnt2 and Wnt11 rescue HDFs activation in the context of Piezo1 knockdown

**Fig. 6:**
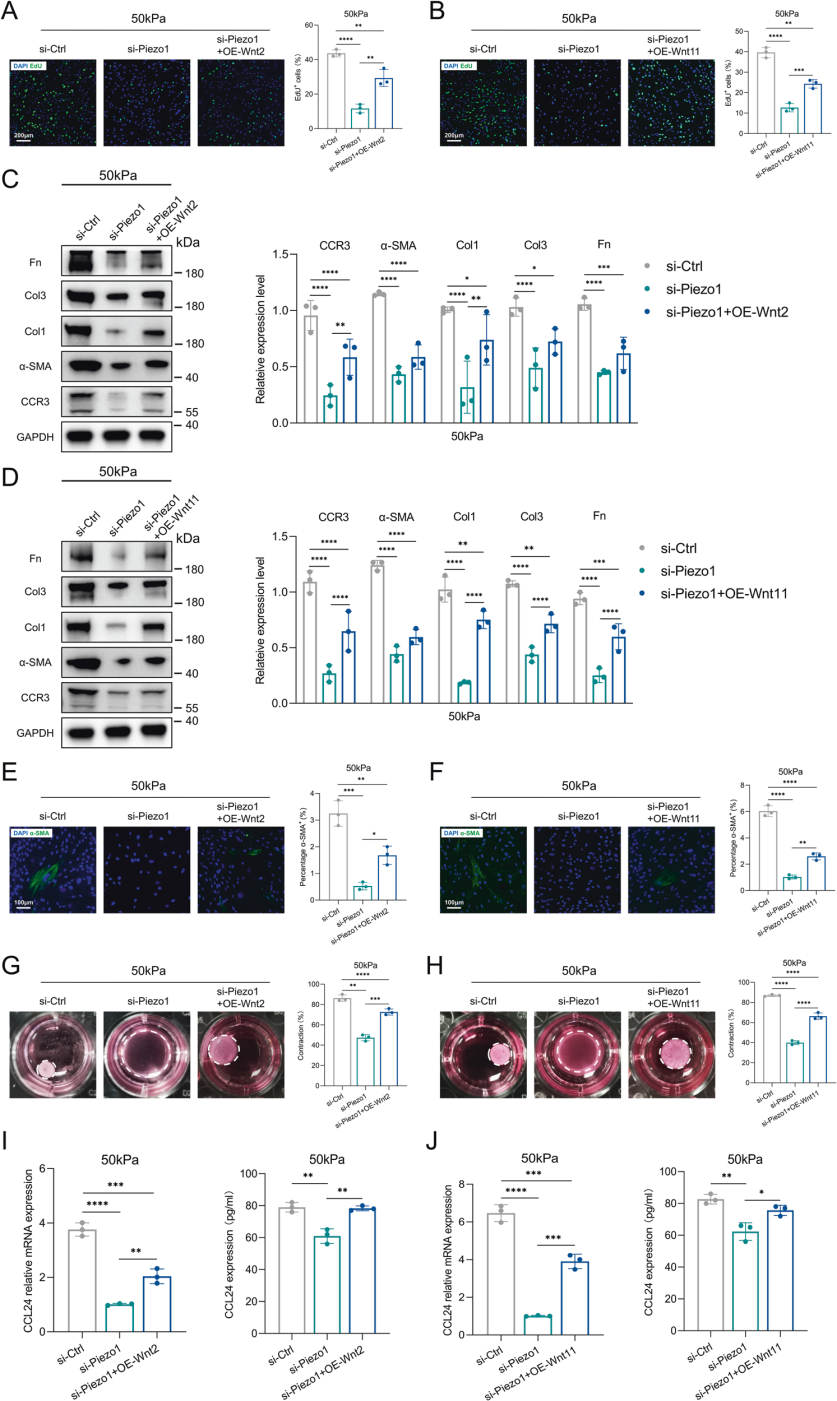
The role of overexpressing Wnt2 and Wnt11 in HDFs activation and inflammation. **A**, **B** HDFs proliferation was detected by EdU immunofluorescent staining and quantitative analysis of the percentage of EdU-positive cells. Scale bar: 200 μm (*n* = 3). **C**, **D** Expression of GAPDH, CCR3, α-SMA, Col1, Col3, and Fn was detected by western blotting and quantitative analysis of protein expression (*n* = 3). **E**, **F** Immunofluorescent staining for α-SMA and quantitative analysis of the percentage of α-SMA positive cells. Scale bar: 100 μm (*n* = 3). **G**, **H** Images and quantitative analysis of fibroblast contraction in three-dimensional collagen lattices (*n* = 3). **I**, **J** CCL24 mRNA expression was detected by RT-PCR and CCL24 concentration in the medium was detected by ELISA (*n* = 3). The results are expressed as the means with SD. “*n*” meaning (Western blot: Statistics were collected with “*n*” independent gel blots; PCR, ELISA, immunostaining and photograph: Statistics were collected with “*n*” independent biological samples). One-way ANOVA is used for all analyses. *P < 0.05, **P < 0.01, ***P < 0.005, ****P < 0.001.

To investigate whether overexpression of Wnt2/Wnt11 in HDFs could rescue Piezo1-loss phenotypes, we overexpressed Wnt2/Wnt11 by virus. Western blot showed that Wnt2/Wnt11 was overexpressed in HDFs (Supplementary Fig. 3). In our assay, Wnt2/Wnt11 overexpression rescued the proliferation of HDFs in Piezo1-loss background (Fig. 6A, B). Furthermore, Wnt2/Wnt11 overexpression also rescued activation of HDFs, including the synthesis of ECM components, α-SMA production and cellular contractility (Fig. 6C–H). As shown by PCR and in vitro ELISA assay, Wnt2/Wnt11 overexpression increased CCL24 release (Fig. 6I, J). Collectively, our data confirmed that Wnt2/Wnt11 served as an important mediator in the Piezo1-CCL24 pathway in the activation of HDFs.

### AAV9-targeting Piezo1 knockdown attenuates skin fibrosis progression and skin stiffness in bleomycin-induced mice skin fibrosis model

**Fig. 7:**
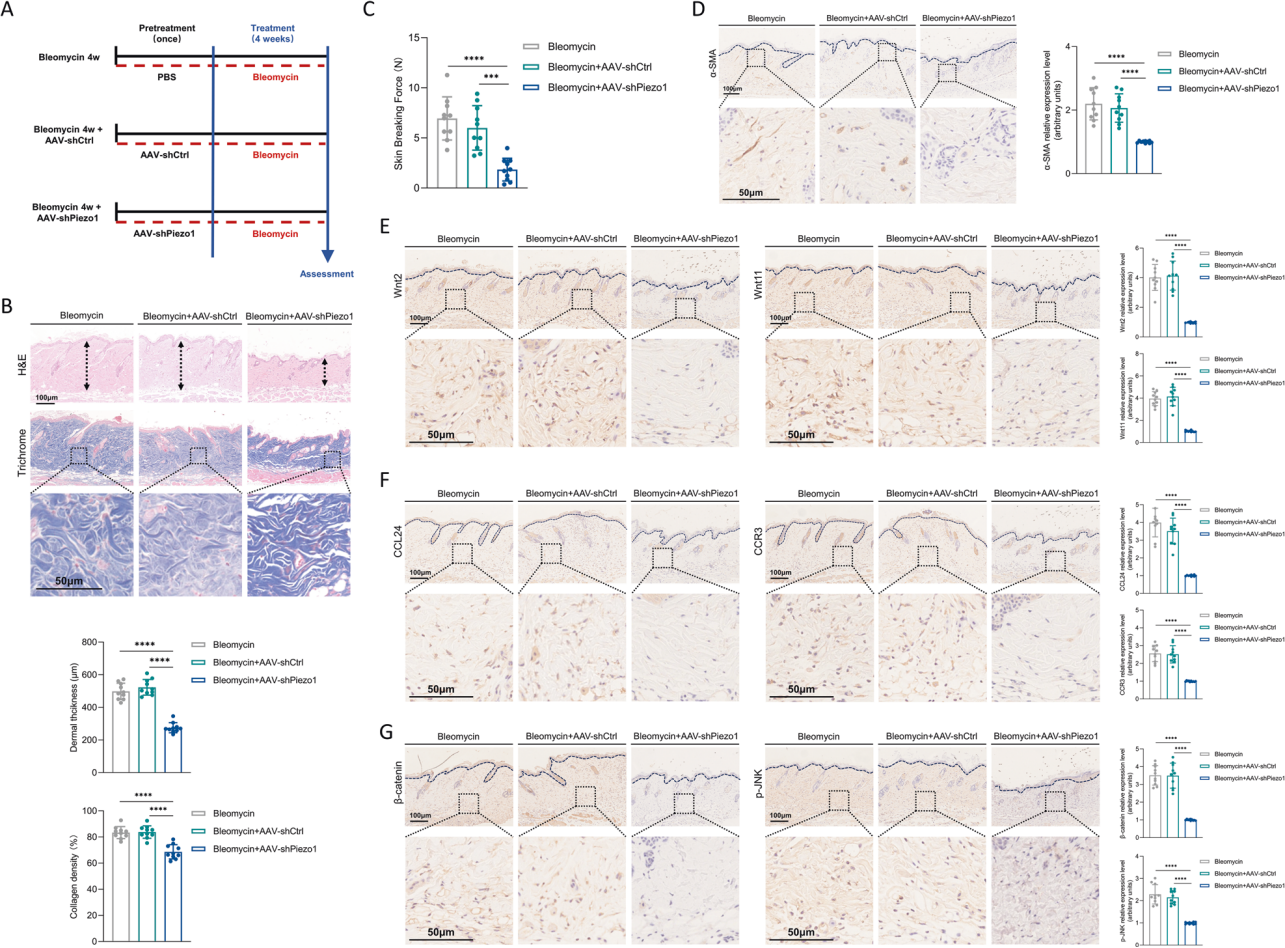
The effect of Intradermal treatment with AAV9-shPiezo1 on mice skin fibrosis progression. **A** Schematic showing the experimental approach. Mice were divided into three groups (PBS-pretreated, AAV9-shCtrl-pretreated, and AAV9-shPiezo1-pretreated) and then exposed to bleomycin-challenged. **B** Representative images and quantitative analysis of H&E and Trichrome staining in three groups. Scale bar:100 μm; Zoom scale bar: 50 μm (*n* = 10). **C** Skin breaking force calculated for three groups. **D** Representative images and quantitative analysis of α-SMA staining in three groups. Scale bar:100 μm; Zoom scale bar: 50 μm (*n* = 10). **E** Representative images and quantitative analysis of Wnt2 and Wnt11 staining in three groups. Scale bar:100 μm (*n* = 10). **F** Representative images and quantitative analysis of CCL24 and CCR3 staining in three groups. Scale bar:100 μm (*n* = 10). **G** Representative images and quantitative analysis of p-JNK and β-catenin staining in three groups. Scale bar:100 μm (*n* = 10). The results are expressed as the means with SD. “*n*” meaning (Histology and immunostaining: Statistics were collected with “*n*” independent biological samples). One-way ANOVA is used for all analysis. ***P < 0.005, ****P < 0.001.

In mechanical stiffness-induced central nervous system ageing, AAV-mediated Piezo1 knockdown significantly improves cellular regenerative capacity [15]. To clarify the critical role of Piezo1 in promoting mechanical stiffness-induced skin fibrosis, we investigated the effects of AAV9-shPiezo1 treatment in the bleomycin-induced mouse skin fibrosis model. We injected AAV9-shPiezo1 and AAV9-shCtrl into the dorsal skin of mice. After three weeks, Piezo1 expression was significantly reduced in AAV9-shRNA-treated mice compared to AAV9-shCtrl-treated mice (Supplementary Fig. 4). Subsequently, skin fibrosis was induced by bleomycin injections for four weeks (Fig. 7A). Importantly, bleomycin-induced fibrosis was significantly less severe in AAV9-shPiezo1-treated mice compared to AAV9-shCtrl-treated mice, with reduced skin thickness and collagen deposition (Fig. 7B). We next determined whether Piezo1 knockdown reduced mechanical stiffness of the skin. AAV9-shPiezo1 treated mice had significantly reduced tensile strength (Fig. 7C). Using immunostaining, we observe that Piezo1 knockdown downregulates the number of α-SMA^+^ myofibroblasts in vivo (Fig. 7D). AAV9-shPiezo1 also reduced the levels of Piezo1 downstream signaling (Wnt2; Wnt11; CCL24; CCR3) (Fig. 7E, F) and Wnt2/Wnt11 downstream signaling (β-catenin; pJNK) (Fig. 7G) in bleomycin-treated mice. Importantly, we performed intradermal administration of recombinant mouse CCL24 in our animal model. We observed that CCL24 injection restored the fibrotic phenotype of Piezo1-deficient mice upon bleomycin stimulation, confirming the key role of the Piezo1-CCL24 axis in skin fibrosis (Supplementary Fig. 5). Taken together, our data confirmed the role of the Piezo1-Wnt2/Wnt11-CCL24 pathway in mechanical stiffness-induced skin fibrosis.

## Discussion

The interplay between mechanical stiffness and biochemical signaling pathways controls the initiation and progression of tissue fibrosis [23]. This process forms a positive feedback loop with increased matrix stiffness and fibroblast activation [24]. In this loop, increased matrix stiffness promotes fibroblast differentiation, then ECM proteins produced by these activated myofibroblasts further upregulate matrix stiffness, completing this amplification loop. Although the effect of the inflammation-mediated positive feedback loop on skin fibrosis has been described [25], the mechanosensitive molecules that detect abnormal matrix stiffness and translate mechanical stiffness into intracellular signaling have not been fully identified.

Piezo1-mediated mechanotransduction is critical for fibrosis progression [26, 27]. We therefore propose a previously unrecognized in vivo role for Piezo1 in promoting skin fibrosis aggression by establishing a positive feedback loop. Piezo1 is overexpressed in myofibroblasts of human and mouse fibrotic skin tissues, suggesting that higher tissue mechanics may trigger Piezo1 expression. Piezo1 expression is also upregulated at higher substrate stiffness in vitro, confirming previous findings that increased tissue mechanics could stimulate Piezo1 expression in different cell types [28–30]. Importantly, our data confirmed that mechanical stiffness could promote the activation of dermal fibroblasts through the Piezo1 channel. In cardiac fibrosis, Piezo1 is involved in matrix stiffness-induced activation of cardiac fibroblasts [24]. This association between Piezo1 expression and fibroblast activation is consistent with increasing matrix stiffening, and Piezo1 upregulation and function may be amplified to exacerbate fibroblast activation. Collectively, this phenomenon suggests that Piezo1-mediated mechanosensation may serve as a general mechanism for dermal fibroblasts to sense and respond to their abnormal tissue mechanics in skin fibrosis.

In several different organ systems, cytokines have been identified as key inducers of fibrosis [31]. Studies have shown that Piezo1 activation results in the secretion of several known cytokines, including FGF1 [32], IL-6 [33], TNF-α [34] and TGF-β [35]. It is possible that in addition to Piezo1-mediated fibroblast activation, Piezo1 may also control fibroblasts activation through cytokine-mediated mechanisms. We therefore investigated whether Piezo1 modulates cytokine signaling in fibroblasts activation and then focused on the downregulation of CCL24 in the context of Piezo1 knockdown. CCL24 is a chemokine that stimulates immune cell trafficking and activation as well as profibrotic activities through the receptor CCR3 [36]. Previous studies have shown that both CCL24 and CCR3 are involved in skin and lung inflammation and fibrosis [37, 38]. CCL24 has been shown to promote collagen production in human lung fibroblasts and plays a critical role in promoting profibrotic effects in idiopathic pulmonary fibrosis (IPF) [39]. Our research has shown that CCL24 promotes the activation of dermal fibroblasts. In recent years, there has been increasing evidence that Piezo1 links physical forces to inflammation in several physiological processes. Piezo1 is activated in response to cyclic pressure and drives c-JUN activation, endothelin-1 overexpression and HIF1α stabilization, facilitating the pro-inflammatory program in mouse lung fibrosis [40]. In aortic valve stenosis, Piezo1 was identified as the main mechanoreceptor responsible for driving monocyte and plasma inflammatory phenotypes [41]. Macrophages lacking Piezo1 also exhibit reduced inflammation and enhanced wound healing responses [16]. Collectively, our results highlight a novel mechanosensory inflammatory axis in which the CCL24-dependent inflammatory pathway plays an important role in Piezo1-mediated dermal fibroblasts activation.

We further elucidate the intracellular signaling Wnt2/Wnt11 that links Piezo1 to CCL24 secretion. Several reports have confirmed that Wnt signaling serves as a downstream mediator of Piezo1. In osteoblast differentiation, Piezo1 activation mediates mechanotransduction via the Wnt/β-catenin pathway [22]. Cyclic mechanical force affects cardiac function-associated protein expressions through the Piezo1/Wnt pathway [42]. Wnt signaling is involved in a variety of physiological processes, and aberrant regulation of this pathway has been implicated in a number of diseases, including cancer and fibrosis [43]. Recent studies have implicated Wnt2 and Wnt11 in the progression of cardiac and pulmonary fibrosis [44–47]. In particular, Wnt2 and Wnt11 expression is increased in skin fibrosis [48], providing new evidence for a potential role of Wnt antagonists in regulating ECM composition in skin fibrosis. Direct regulation of Wnt signaling by mechanical cues has been demonstrated. Extracellular mechanical cues regulate intestinal organoid growth by increasing Wnt/β-catenin signaling [49]. Mechanical features of the microenvironment influence the tissue-specific differentiation of stem cells through an enhanced Wnt-dependent mechanism [50]. Strikingly, in mechanical force-mediated tissue fibrosis, Wnt11 may be a novel therapeutic target for the prevention of cardiac fibrosis under pressure overload [45]. Therefore, together with our findings, we highlight the important role of Wnt2/Wnt11 in linking Piezo1 and aberrant tissue mechanics mediated fibrosis. In addition, recent evidence has shed light on the cellular processes by which Wnt signaling and conventional inflammatory cytokines control disease progression [51]. Cancer cells lacking p53 induced the secretion of Wnt ligands, which stimulate tumor-associated macrophages to produce IL-1β, thereby dictating pro-metastatic systemic inflammation [52]. In a novel transgenic mouse model of overload proteinuria, activation of Wnt/β-catenin signaling enhances intrarenal inflammation via the TLR-4/NLRP-3 inflammasome axis [53]. After Wnt2 or Wnt11 knockdown, both CCL24 and CCR3 expression were downregulated in fibroblasts cultured on higher substrate stiffness. Taken together, these results suggest that mechanical stiffness regulates fibroblast activation through the Piezo1-Wnt2/Wnt11-CCL24 inflammatory pathway.

Gene therapy has been increasingly successful owing to enhanced molecular understanding of human disease and the development of gene delivery technologies [54]. Among these technologies, adeno-associated virus (AAV) delivery vectors have proven to be safe and effective [55]. AAV also holds promise as a useful vector for skin-directed gene therapy in chronic wound healing [56, 57]. In our research, AAV-targeted Piezo1 knockdown effectively ameliorates the progression of skin fibrosis and skin rigidity in mice. Similarly, AAV delivery shows therapeutic potential in the treatment of fibrotic diseases. For example, AAV-mediated silencing of CD47 [58], KDM4D [59] and nestin [60] has been used as a strategy to reduce liver fibrosis. Localized AAV-mediated inhibitor of differentiation 3 gene therapy in rabbit eyes abolished corneal fibrosis in vivo [61]. Overall, our findings may provide a new genetic therapeutic avenue for the treatment of skin fibrosis and, more importantly, mechano-based therapeutics targeting Piezo1 showed promising results in breaking the positive feedback loop between cell mechanotransduction and aberrant tissue mechanics.

There are several limitations to this study. Indeed, it has been reported that Piezo1, Wnt2/Wnt11 and CCL24 play a role in the progression of skin fibrosis. Hence, the level of research innovation is moderate. Our previous reports first showed that Piezo1 plays an important role in mechanical force-induced skin fibrosis [17, 62]. However, the mechanism between Piezo1 and skin fibrosis is not clear. Since inflammation serves as a driver in the progression of skin fibrosis [63], we speculate whether Piezo1 links cytokines/chemokines in skin fibrosis. Using cytokine antibody arrays, we selected CCL24, a chemokine strongly associated with tissue fibrosis. We then identified Wnt2/Wnt11 as the link between Piezo1 and CCL24. Thus, we show that Piezo1-Wnt2/Wnt11-CCL24 is a novel positive feedback loop, especially in mechanical force-induced skin fibrosis.

**Fig. 8:**
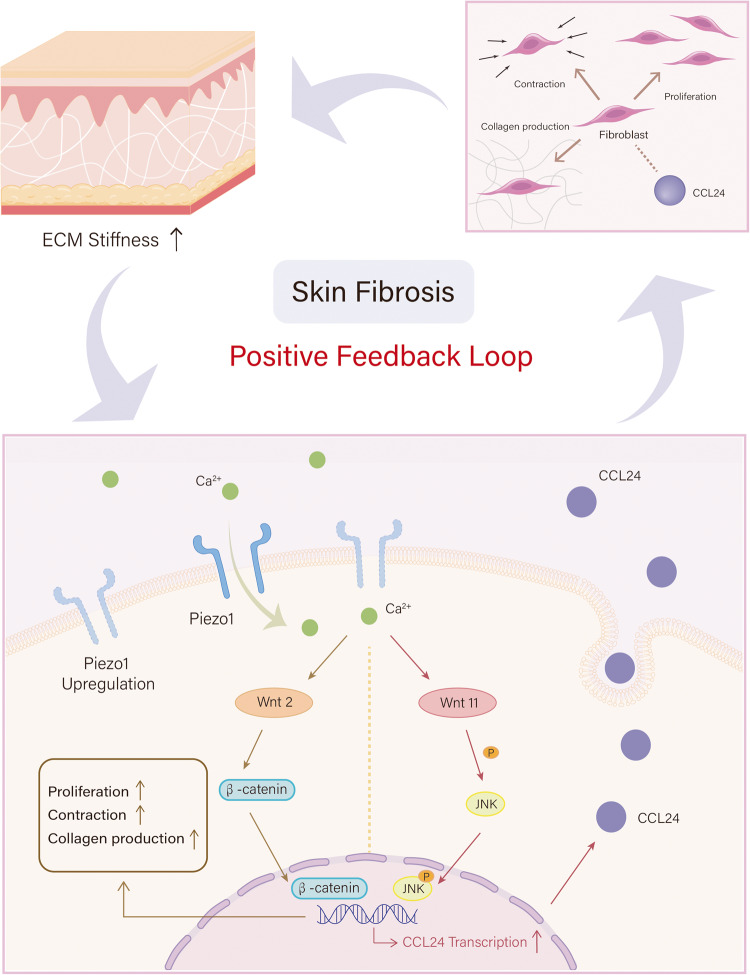
A proposed model showing the positive feedback loop between Piezo1-Wnt2/Wnt11-CCL24 pathway and aberrant tissue mechanics in promoting skin fibrosis. During skin fibrosis progression, skin tissue stiffening provides a mechanical microenvironment to activate Piezo1. On the one hand, Piezo1 regulates dermal fibroblasts activation through Wnt2/Wnt11 signaling. On the other hand, CCL24 secretion through Piezo1-Wnt2/Wnt11 pathway could directly activate dermal fibroblasts. In addition, tissue stiffening (collagen over-production by dermal fibroblasts activation) in the skin further increases the protein level of Piezo1, which in turn elevates the mechanotransduction capacity of dermal fibroblasts. These processes form a positive feedback loop between dermal fibroblasts mechanotransduction and aberrant tissue mechanics to promote skin fibrosis progression.

Based on these findings, we propose a model for mechanical stiffness-mediated skin fibrosis in which aberrant tissue mechanics activates both dermal fibroblast activation and CCL24 via Piezo1-Wnt2/Wnt11 to maintain a positive feedback loop (Fig. 8). These studies provide a framework for understanding how mechanical stiffness triggers inflammatory responses to induce skin fibrosis progression. More broadly, these results suggest that gene therapy targeting Piezo1 could effectively uncouple mechanical stiffness from inflammation and fibrosis and may prove successful in various human diseases.

## Materials and methods

### Patient samples

Fresh human normal skin, hypertrophic scar, and keloid tissues (Supplementary Table 1) were collected from Shanghai Ninth People’s Hospital with ethics approval from the local Human Research Ethics Committee of Shanghai Jiao Tong University School of Medicine in accordance with the principles of the Declaration of Helsinki. Upon collection, samples were directly fixed with PFA and then processed for the following analyses.

### Cell isolation and culture

The normal human skin tissues or neonatal mouse dorsal skin were placed in a 0.2% Dispase II solution (Sigma‐Aldrich, St Louis, MO, USA) overnight at 4 °C. The next day, the dermal skin was digested with 0.25% collagenase IV solution (Sigma‐Aldrich) at 37 °C for 2 h. After filtration, centrifugation, and resuspension, the human dermal fibroblasts (HDFs) or mouse dermal fibroblasts (MDFs) were cultured in Dulbecco’s Modified Eagle Medium (DMEM) (Gibco Life Technologies, Grand Island, NY, USA) supplemented with 10% fetal bovine serum (FBS) (Gibco Life Technologies) and 1% penicillin/streptomycin (Gibco Life Technologies) at 37 °C and 5% CO_2_. Passage 3–5 cells were used for experiments.

### Quantitative real-time reverse transcriptase-PCR (RT-PCR)

TRIzol reagent was used to extract total RNA. The RNA was reverse transcribed into cDNA using RevertAid Reverse Transcriptase (Thermo Fisher Scientific, Waltham, MA, USA) according to the manufacturer’s instructions. Real-time PCR was performed with SYBR Premix EX Taq (Takara, Dalian, China) and LightCycler 480 System (Roche, Indianapolis, IN, USA). The primers used in our study are shown in Supplementary Table 2.

### Histology and immunohistochemistry

Samples were fixed with 4% paraformaldehyde overnight, then embedded in paraffin. 5 mm sections were processed and stained with hematoxylin and eosin (H&E) and Masson’s Trichrome Stain Kit following protocol (Solarbio, Beijing, China).

We used the following antibodies to investigate protein expression in these samples by immunohistochemistry: Piezo1 (ab128245, 1:100, Abcam, Cambridge, UK), CCL24 (22306-1-AP, 1:200, Proteintech, Wuhan, China), CCR3 (ab32512, 1:100, Abcam), Wnt2 (A5864, 1:200, Abclonal, Wuhan, China), Wnt11 (ab31962, 1:200, Abcam), α-SMA (#19245, 1:200, Cell Signaling Technology, Danvers, USA), p-JNK (sc-6254, 1:100, Santa Cruz Biotechnology, Dallas, Texas, USA), β-catenin(#8480, 1:100, CST). Images were captured using a Nikon Eclipse E800 microscope (Nikon, Melville, NY, USA).

### Immunofluorescence

Cell samples were fixed with 4% PFA for 15 min at RT. Tissues were fixed with 4% PFA overnight at RT. Tissue samples were embedded in paraffin and sliced into 5 mm sections. Samples were incubated with following antibodies: Piezo1 (ab128245, 1:100, Abcam), α-SMA (ab7817, 1:100, Abcam), CCL24 (22306-1-AP, 1:200, Proteintech), CCR3 (ab32512, 1:100, Abcam), Wnt2 (A5864, 1:200, Abclonal, Wuhan, China), Wnt11 (ab31962, 1:200, Abcam), β-catenin(#8480, 1:100, CST), Alexa Fluor 594 goat anti-rabbit secondary antibody (Jackson lab, 125369, 1:200) and Alexa Fluor 488 goat anti-mouse secondary antibody (Jackson lab, 133384, 1:200). Images were visualized using a Nikon Eclipse E800 microscope (Nikon, Melville, NY, USA).

### Polyacrylamide hydrogel-coated plates

To test the effect of matrix stiffness on HDFs behaviors, cells were seeded on collagen-coated polyacrylamide hydrogels with different stiffnesses (2 kPa and 50 kPa) for 7 days. 2 kPa plates mimic the elastic modulus of human normal skin tissues and 50 kPa plates mimic the elastic modulus of human fibrotic skin tissues according to previous report [64].

#### 5-ethynyl-20-deoxyuridine (EdU) staining

EdU staining was detected using the Click-iT EdU Imaging Kits (Invitrogen) according to the manufacturer’s protocol. Briefly, cells were incubated with 10 mM EdU for 1 h before fixation, permeabilization, and EdU staining. The nuclear was stained with DAPI (Sigma-Aldrich, St. Louis, MO).

#### Apoptosis assay

The Annexin V Apoptosis Detection Kit (BD Biosciences, San Jose, CA, USA) was utilized to assess cellular apoptosis. HDFs were washed, centrifuged, and then resuspended in PBS buffer containing annexin V-FITC. The cells were incubated at room temperature for 15 min. The samples were analyzed with a Gallios flow cytometer (Beckman Coulter, Brea, CA, USA).

### siRNA and transfection

For gene silencing, HDFs were transfected with 100 nM siRNA by using Lipofectamine RNAiMAX reagent (Invitrogen, Carlsbad, CA, USA) according to the manufacturer’s protocol. The sequences were as follows: Piezo1-siRNA, 5′-AGAAGAAGAUCGU CAAGUATT-3′ (sense) and 5′-UACUUGACGAUCUUC UUCUTT-3′ (antisense), Wnt2-siRNA, 5′-GUUCCUGUGAUCCAAAGAATT-3′ (sense) and 5′-UUCUUUGGAUCACAGGAACTT-3′ (antisense), Wnt11-siRNA, 5′-GAACUCGUCUAUCUGCAGATT-3′ (sense) and 5′-UCUGCAGAUAGACGAGUUCTT-3′ (antisense), Ctrl (Control)-siRNA, 5′-GUGAGCGUCUAUAUACCAUTT-3′ (sense) and 5′-AUGGUAUAUAGACGCUCACTT-3′ (antisense).

### Collagen gel contraction assay

HDFs were resuspended in collagen gel (Shengyou, Hangzhou, China). Suspension was plated in each well of a 24-well plate. The plates were incubated at 37 °C for 15 min to allow collagen gel polymerization. After gel polymerization, 1 ml DMEM supplemented with 10% FBS was added to each well. The gels were photographed post 48 h.

### Cytokine antibody arrays

The levels of 40 inflammatory cytokines in the supernatants of HDFs were assayed using the Human Inflammation Array Q3 based on the manufacturer’s instructions (RayBiotech Inc., Norcross, GA, USA).

### Enzyme-linked immunosorbent assay (ELISA)

After the desired treatment, the culture medium was collected and centrifuged at 2500 rpm for 10 min. The supernatants were collected and the concentration of cytokines CCL24 was measured by ELISA kits from the R&D system (Minneapolis, MN, USA) in accordance with the instruction manual.

### CCL24/Eotaxin-2 protein treatment

Recombinant human CCL24/Eotaxin-2 protein (HY-P7161, MedChemExpress, Shanghai, China) and Recombinant mouse CCL24/Eotaxin-2 protein (HY-P7760, MedChemExpress, Shanghai, China) was purchased and dissolved in PBS solution (Human CCL24 concentration: 5 ng/ml, 10 ng/ml, 15 ng/ml; Mouse CCL24 concentration: 10 ng/ml) according to the manufacturer’s instructions.

### RNA sequencing

For RNA-seq analysis, *N* = 3 biological replicates were sequenced per experimental group (HDFs were pretreated with Piezo1-siRNA or NC-siRNA, then cultured in 50kPa plates for 7 days). Total RNA was extracted using the Trizol reagent. RNA integrity was assessed using the Agilent 2100 Bioanalyzer (Agilent Technologies, Santa Clara, CA, USA). Samples with an RNA Integrity Number ≥ 7 were processed for the subsequent analysis. The TruSeq Stranded mRNA LT Sample Prep Kit (Illumina, San Diego, CA, USA) was used to construct cDNA libraries. Differentially expressed genes (DEGs) were done using the R package DEseq2 (v 1.6.3) [65]. *P* < 0.05 and fold change >2 or fold change <0.5 was set as the threshold for a significant differential expression pattern. Hierarchical cluster analysis of DEGs was performed to examine transcript expression patterns. Gene Ontology enrichment analysis and KEGG pathway enrichment analysis of DEGs were performed using R based on the hypergeometric distribution.

### Western blotting

Cells were lysed with ice-cold RIPA buffer and centrifuged at 13,000 *g* for 15 min at 4 °C. 10 μg of protein lysate (concentration determined by a bicinchoninic acid assay) was run on a 10% SDS-PAGE gel at 150 V for 1 h and then transferred to nitrocellulose filter membranes (Millipore, Bedford, MA). After blocking with 5% BSA at RT for 1 h, the membranes were incubated with the following primary antibodies: Piezo1 (ab128245, 1:1000, Abcam), Collagen I (ab34710, 1:500, Abcam), Collagen III (ab7778, 1:500, Abcam), fibronectin (ab2413, 1:1000, Abcam), α-SMA (#19245, 1:1000, CST), GAPDH (#5174, 1:1000, CST), CCR3 (ab32512, 1:1000, Abcam), Wnt2 (A5864, 1:1000, Abclonal), Wnt11 (bs-8568R, 1:1000, Bioss), JNK (#9252, 1:1000, CST), p-JNK (#4668, 1:1000, CST) at 4 °C overnight. The next day, membranes were incubated with an Anti-rabbit IgG, HRP-linked Antibody (7074 S, 1:1000, CST) at room temperature for 1 h and then washed with tris-buffered saline with 0.1% Tween 20 three times for 10 min. Quantitative analysis was performed on the immunoreactive bands with ImageJ software.

### Animal models of fibrosis

Briefly, 8-week-old C57/BL6 mice (SLAC Laboratory Animal, Shanghai, China) were anesthetized. 100 μL of either AAV9-shPiezo1 or AAV9-shCtrl was injected subcutaneously into the dorsal skin of the mice 21 days before bleomycin injection. In the bleomycin-induced skin fibrosis model, skin fibrosis was induced by subcutaneous injection of bleomycin (Yeasen Biotechnology, Shanghai, China) for 4 weeks. All animal procedures were performed in accordance with the guidelines of the Animal Care and Use Committee of the School of Medicine, Shanghai Jiao Tong University.

### Adeno-associated virus (AAV) vector construction

The construction of the vector carrying the shRNA (against Piezo1 and no-targeting scramble) and its packaging into AAV-9 were carried out by Vector Builder (vectorbuilder.com) at final titer of AAV9 to >1.0 × 10^12^ GC/ml. The interference sequences were as follows: AAV9-shCtrl: 5′- CCTAAGGTTAAGTCGCCCTCG-3′, AAV9-shPiezo1: 5′- CTGCTATCAGACACCATTTAT-3′. 100 μL (1.0 × 10^11^ GC/ml) of AAV9-shPiezo1 or AAV9-shCtrl was injected into the subcutaneous of mice dorsal skin. After 21 days, mice dorsal skin was collected and performed immunostaining analysis of Piezo1 to confirm whether the target gene has been knocked down.

### Statistical analysis

A two-tailed Student’s *t*-test was used for comparisons between two groups. One-way ANOVA was used for comparing multiple groups. *P* < 0.05 was considered to indicate a significant difference. At least three independent replicates were used for each experiment. Results are expressed as the means ± SD.

### Supplementary information

Reproducibility checklist

Supplemental materials

Full-length western blots

## Data Availability

All data generated or analyzed during this study are available from the corresponding author upon reasonable request.
