# Supplementary material for: Mechanical stiffness promotes skin fibrosis via Piezo1-Wnt2/Wnt11-CCL24 positive feedback loop
Source: Cell Death Dis. 2024 Jan 24;15(1):84. doi: 10.1038/s41419-024-06466-3 (PMC10808102; doi:10.1038/s41419-024-06466-3)

**Supporting Information**

**1. Supplementary Table 1. Patient information**

| Order | Sex | Age (years) | Location |
| --- | --- | --- | --- |
| Hypertrophic scar-1 | Male | 5 | Face |
| Hypertrophic scar-2 | Male | 40 | Neck |
| Hypertrophic scar-3 | Female | 25 | Face |
| Hypertrophic scar-4 | Female | 36 | Neck |
| Hypertrophic scar-5 | Male | 24 | Hand |
| Hypertrophic scar-6 | Female | 20 | Face |
| Hypertrophic scar-7 | Male | 54 | Hand |
| Hypertrophic scar-8 | Female | 32 | Face |
| Keloid-1 | Male | 15 | Chest |
| Keloid-2 | Female | 35 | Chest |
| Keloid-3 | Female | 36 | Abdomen |
| Keloid-4 | Male | 42 | Chest |
| Keloid-5 | Male | 32 | Arm |
| Keloid-6 | Female | 35 | Chest |
| Keloid-7 | Male | 27 | Abdomen |
| Keloid-8 | Female | 30 | Chest |

**2. Supplementary Table 2. Primer list**

| Human Gene | Forward Primer 5’🡪3’ | Reverse Primer 5’🡪3’ |
| --- | --- | --- |
| PIEZO1 | AGCCGAGAGACAGAGAAGAAAT | AAGAGCAGTGGGAACCAGAT |
| PIEZO2 | CATTACCCCACTGATGAGAG | GTTGCCGTTCACAGTCAC |
| TRPV1 | TTGGGGGTGTTGGTGTTT | TGATCTTCTGGAGACTGTGATTG |
| TRPV4 | CGAGGTCATTACGCTCTTCAC | TCACTCCAGGGCATTTCTTC |
| TRPA1 | ACTCTCTAAAGGTGCCCAAGTA | CATCGTTGTCTTCATCCATTAC |
| TRPC1 | TTGGGGGTGTTGGTGTTT | TGATCTTCTGGAGACTGTGATTG |
| CCL24 | ACATCATCCCTACGGGCTCT | CTTGGGGTCGCCACAGAAC |
| GAPDH | GGGAAGGTGAAGGTCGGAGT | GGGGTCATTGATGGCAACA |
| Mouse Gene | Forward Primer 5’🡪3’ | Reverse Primer 5’🡪3’ |
| PIEZO1 | CCACCTTGCCTTTCAGATATG | CAGCCTCGTAACCCCTATGT |
| PIEZO2 | CCGTGTTCCAGTTCATCA | CCTCCTGTTGCGAATCAT |
| TRPV1 | CTTCAAGGCTGTCTTCATCAT | CACTTCAGGAAACTCTTCTCTGT |
| TRPV4 | ACCTGCTGCTTCTCAAGTGTT | CGGATGATGTGCTGAAAGAC |
| TRPA1 | AAATCCAAACCTCCGAAATA | TGTTCCCATTCTCTCCTTCTA |
| TRPC1 | GAACAGCAAAGCAATGACAC | AGGAGAAGATGTACCAGAACAGA |
| GAPDH | CCCGTAGACAAAATGGTGAA | TGCCGTGAGTGGAGTCATAC |

**3. Supplementary Figure**

**
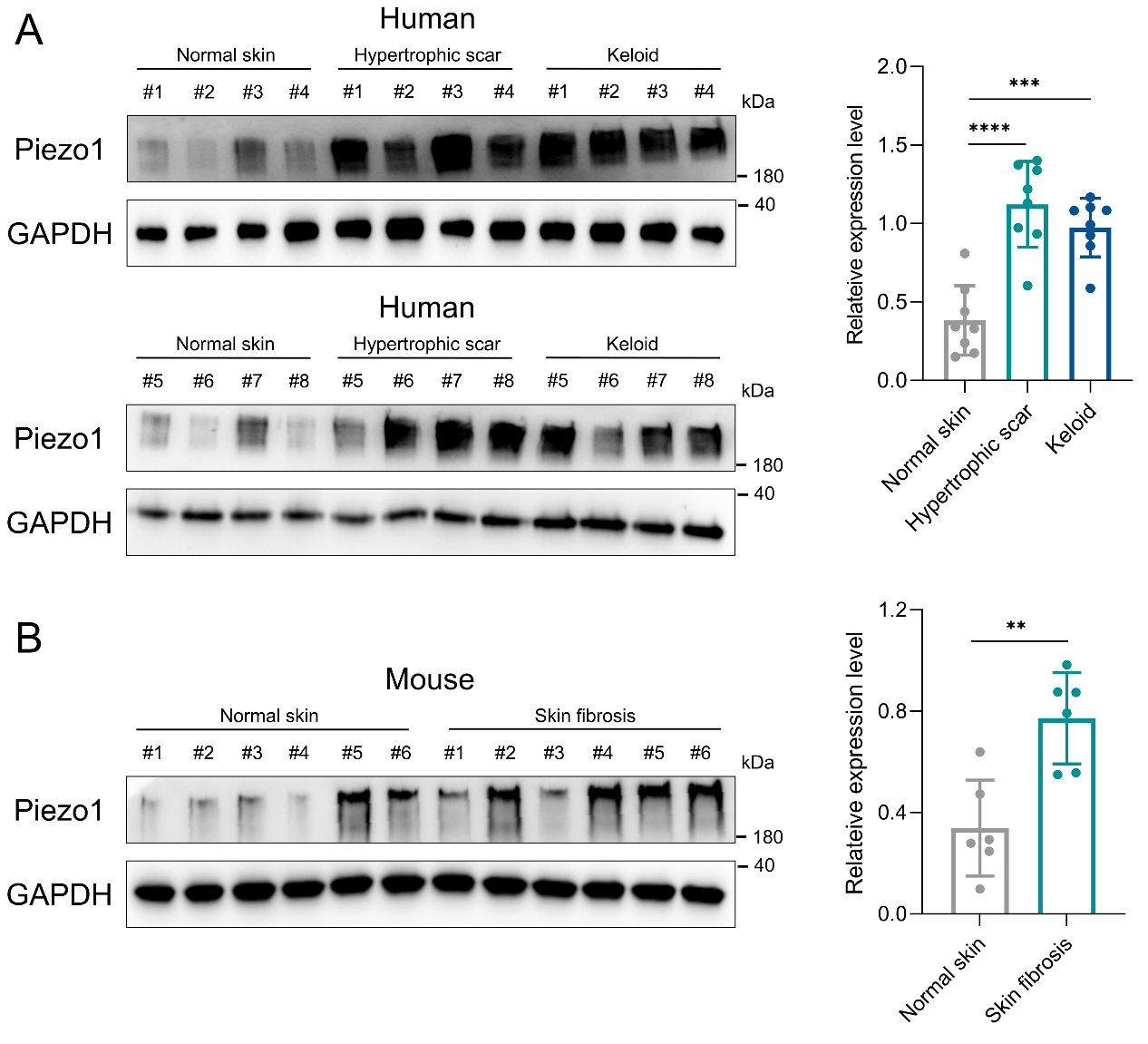
**

Figure legend

Supplementary Figure 1. Piezo1 expression in human and mouse tissues

(A) Expression of GAPDH and Piezo1 in human tissues (Normal skin, Hypertrophic scar and keloid) was detected by western blotting and quantitative analysis of protein expression. (n=8). (B) Expression of GAPDH and Piezo1 in mouse tissues (Normal skin and skin fibrosis) was detected by western blotting and quantitative analysis of protein expression. (n=6). The results are expressed as the means with SD. “n” meaning (Western blot: Statistics were collected with “n” independent patient samples per group or independent mouse tissues per group). Two-tailed t-test and One-way ANOVA is used for all analysis. **P < 0.01, ***P < 0.005, ****P < 0.001.


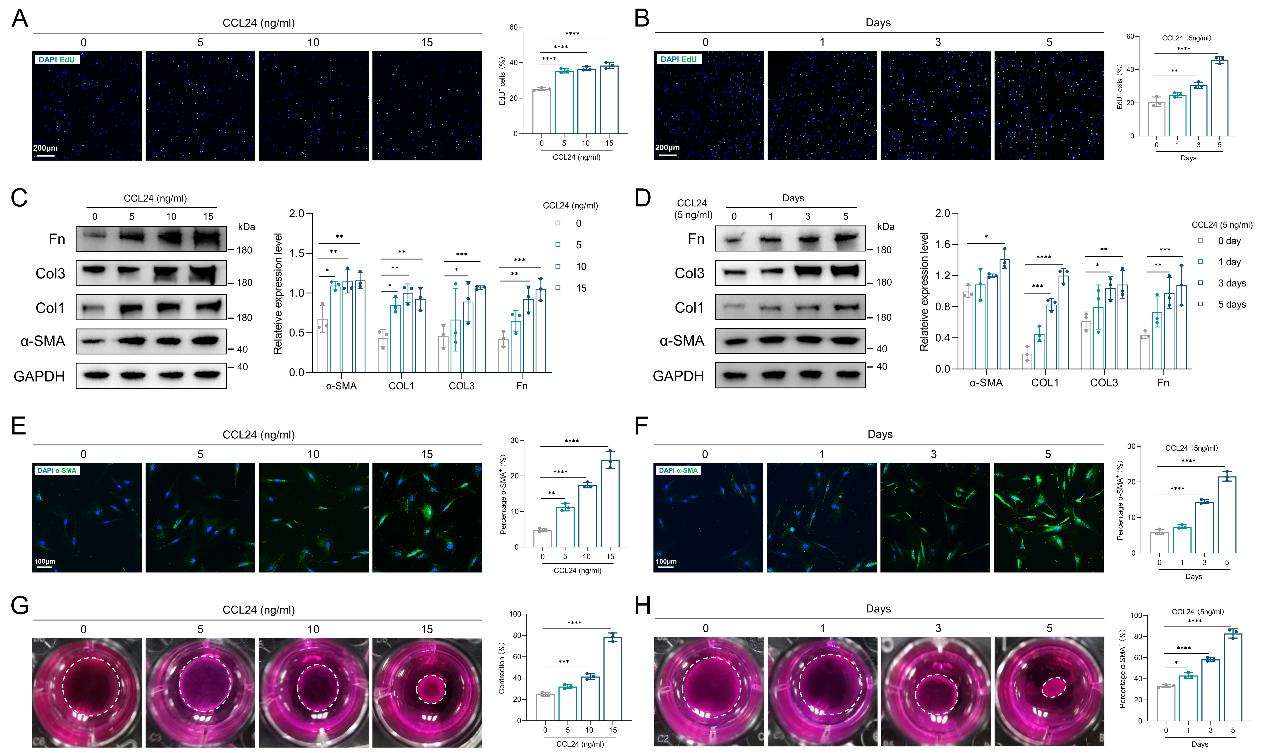


Figure legend

Supplementary Figure 2. The role of CCL24 in HDFs activation

(A) HDFs proliferation was detected by EdU immunofluorescent staining and quantitative analysis of the percentage of EdU positive cells. HDFs were incubated with CCL24 in different concentration for 24h. Scale bar: 200μm (n=3). (B) HDFs proliferation was detected by EdU immunofluorescent staining and quantitative analysis of the percentage of EdU positive cells. HDFs were incubated with CCL24 (5ng/ml) over different periods of time. Scale bar: 200μm (n=3). (C) Expression of GAPDH, α-SMA, Col1, Col3 and Fn was detected by western blotting and quantitative analysis of protein expression. HDFs were incubated with CCL24 in different concentration for 24h (n=3). (D) Expression of GAPDH, α-SMA, Col1, Col3 and Fn was detected by western blotting and quantitative analysis of protein expression. HDFs were incubated with CCL24 (5ng/ml) over different periods of time (n=3). (E) Immunofluorescent staining for α-SMA and quantitative analysis of the percentage of α-SMA positive cells. HDFs were incubated with CCL24 in different concentration for 24h. Scale bar: 100μm (n=3). (F) Immunofluorescent staining for α-SMA and quantitative analysis of the percentage of α-SMA positive cells. HDFs were incubated with CCL24 (5ng/ml) over different periods of time. Scale bar: 100μm (n=3). (G) Images and quantitative analysis of fibroblast contraction in three-dimensional collagen lattices. HDFs were incubated with CCL24 in different concentration for 24h (n=3). (H) Images and quantitative analysis of fibroblast contraction in three-dimensional collagen lattices. HDFs were incubated with CCL24 (5ng/ml) over different periods of time (n=3). The results are expressed as the means with SD. “n” meaning (Western blot: Statistics were collected with “n” independent gel blots; immunostaining and photograph: Statistics were collected with “n” independent biological samples). One-way ANOVA is used for all analysis. *P < 0. 05, **P < 0.01, ***P < 0.005, ****P < 0.001.


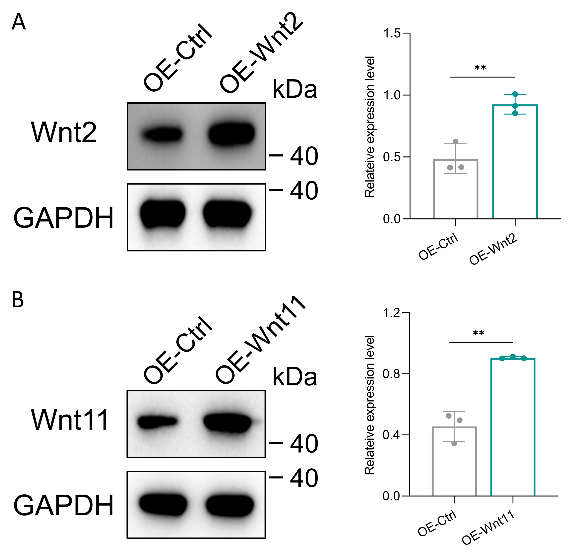


**Figure legend**

**Supplementary Figure 3. Wnt2/Wnt11 overexpression in HDFs.**

(A) Expression of Wnt2 and GAPDH was detected by western blotting and quantitative analysis of protein expression (n=3). (B) Expression of Wnt11 and GAPDH was detected by western blotting and quantitative analysis of protein expression (n=3). “n” meaning (Immunostaining: Statistics were collected with “n” independent biological samples). Two-tailed t-test is used for all analysis. **P < 0.01.


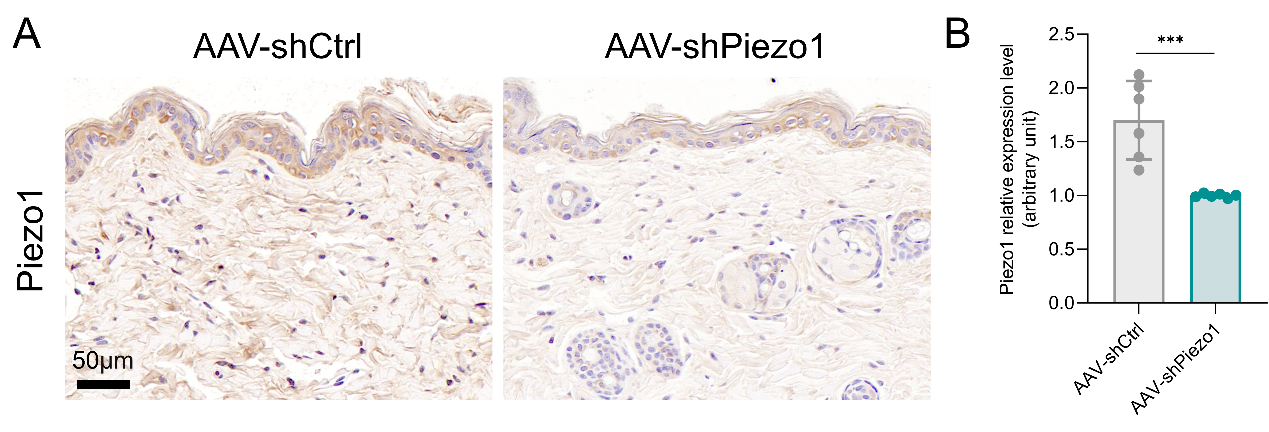


**Figure legend**

**Supplementary Figure 4. Validation of Piezo1 knockdown in mouse skin**

(A) Representative images of Piezo1 staining. (B) Quantitative analysis of Piezo1 staining (n = 6). Scale Bar: 50µm, “n” meaning (Immunostaining: Statistics were collected with “n” independent biological samples). Two-tailed t-test is used for all analysis. ***P < 0.005.


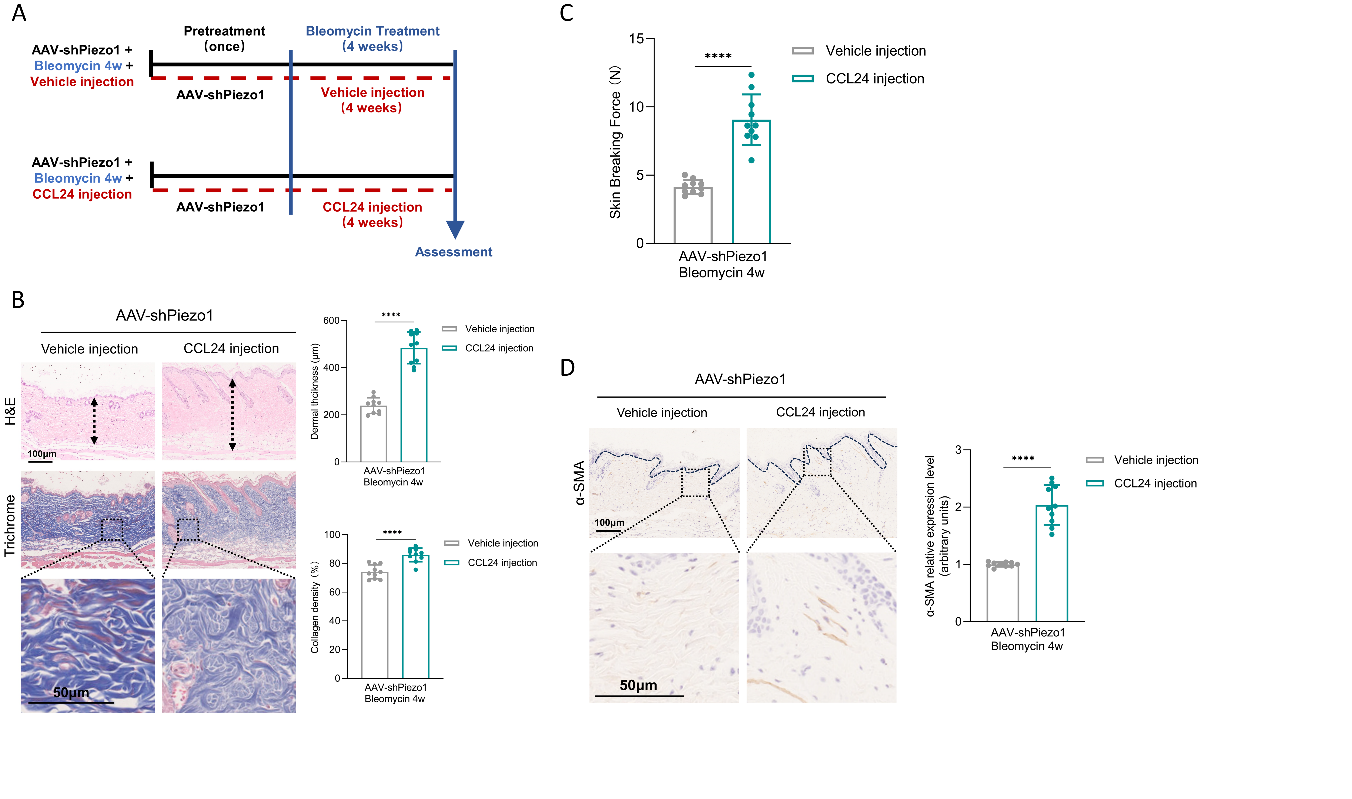


**Figure legend**

**Supplementary Figure 5. CCL24 injection in mice skin fibrosis progression in the context of Piezo1 knockdown**

(A) Schematic showing experimental approach. Mice (AAV9-shPiezo1-pretreated) were divided into 2 groups (CCL24 injection or not) and then exposed to bleomycin challenged. (B) Representative images and quantitative analysis of H&E and Trichrome staining in 2 groups. Scale bar:100μm; Zoom scale bar: 50μm (n=10). (C) Skin breaking force calculated for 2 groups. (D) Representative images and quantitative analysis of α-SMA staining in 2 groups. Scale bar:100μm; Zoom scale bar: 50μm (n=10). The results are expressed as the means with SD. “n” meaning (Histology and immunostaining: Statistics were collected with “n” independent biological samples). Two-tailed t-test is used for all analysis. ****P < 0.001.


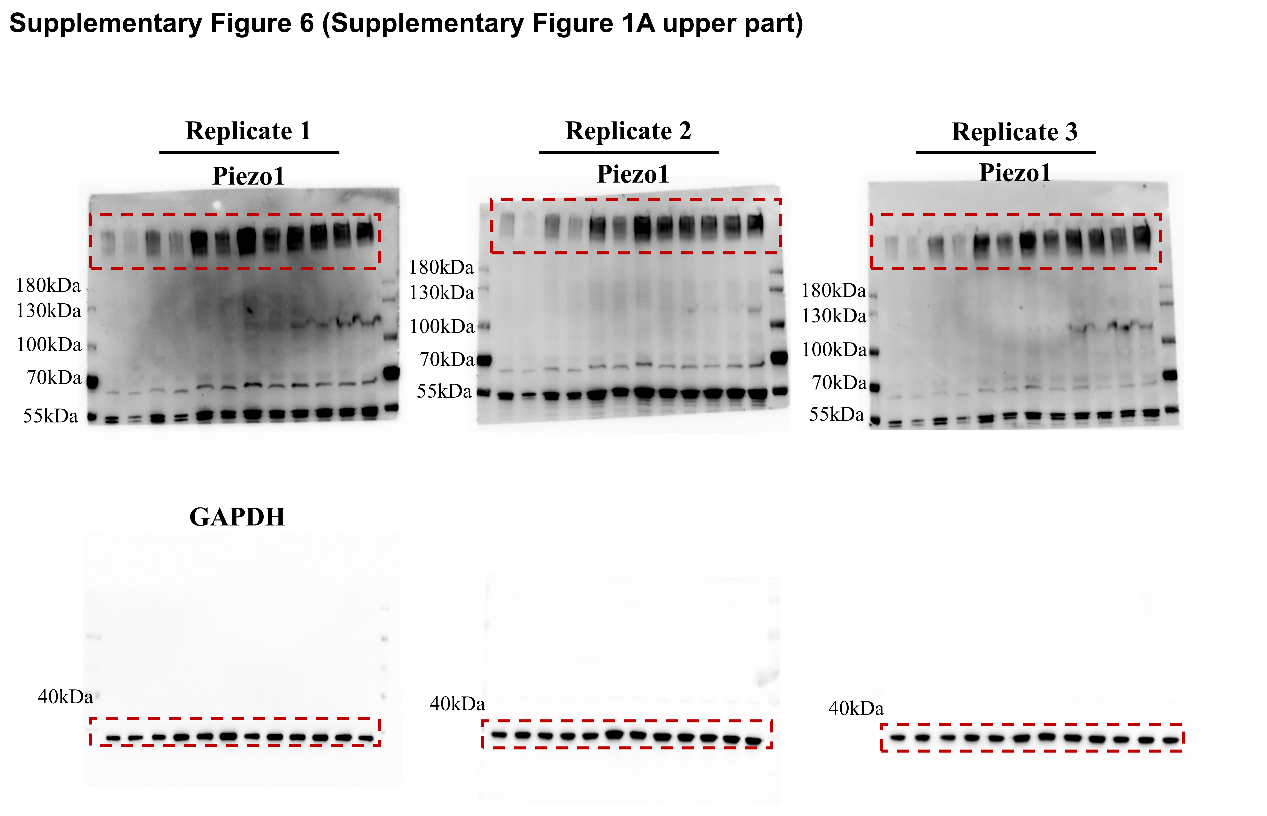

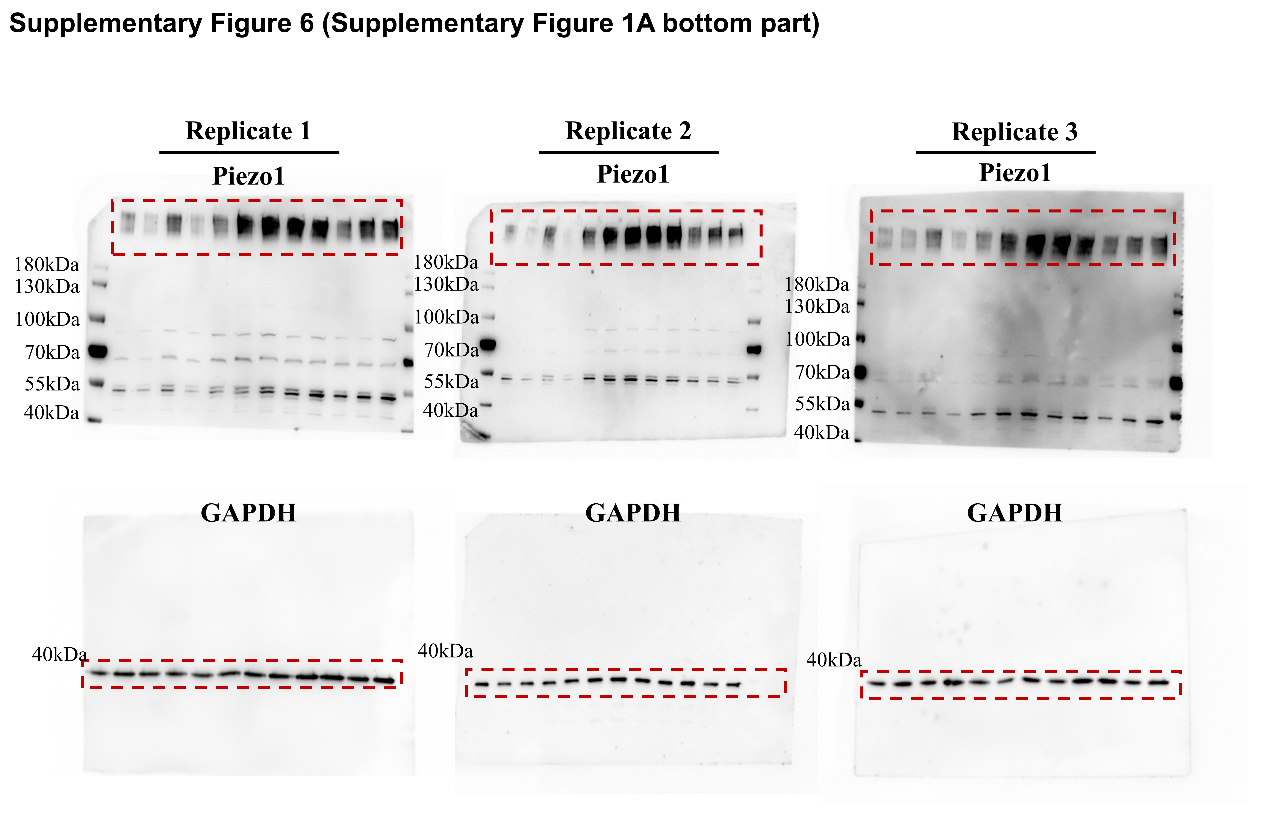

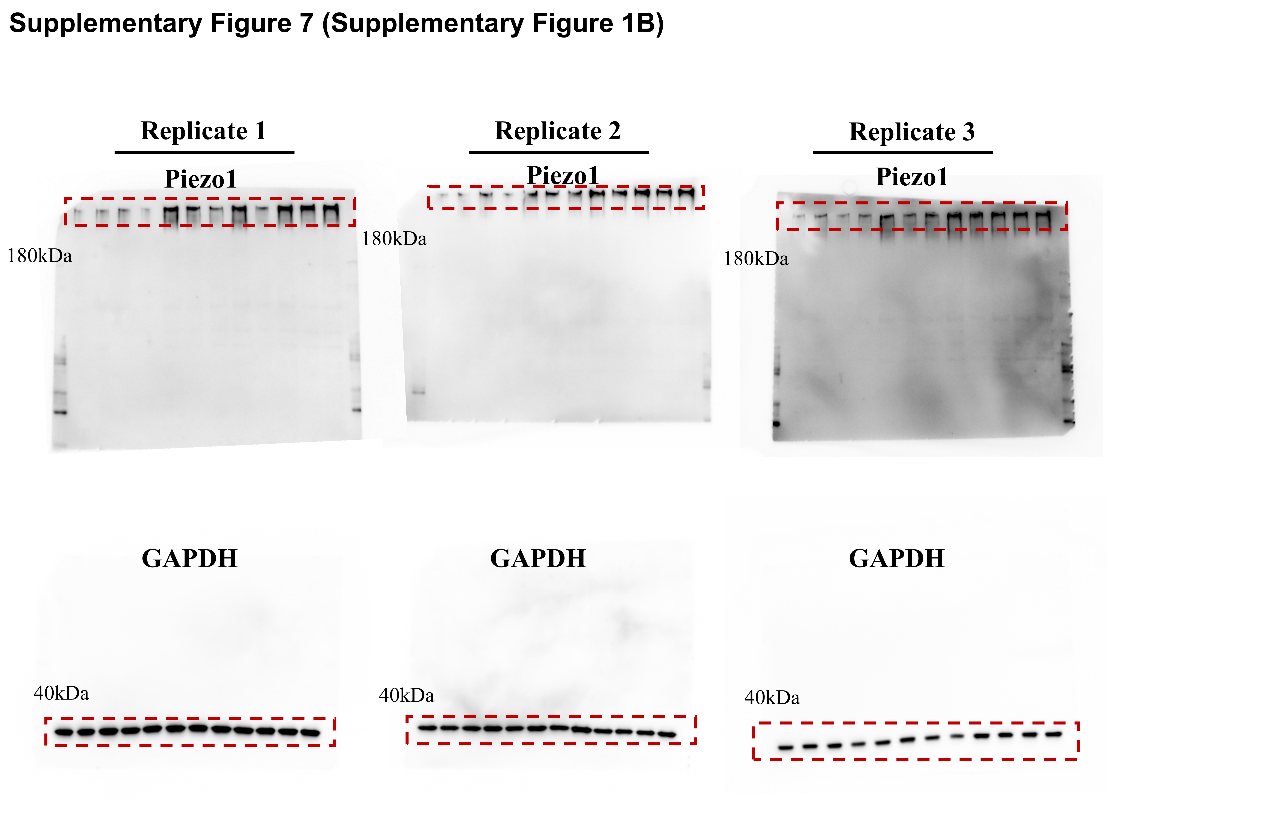

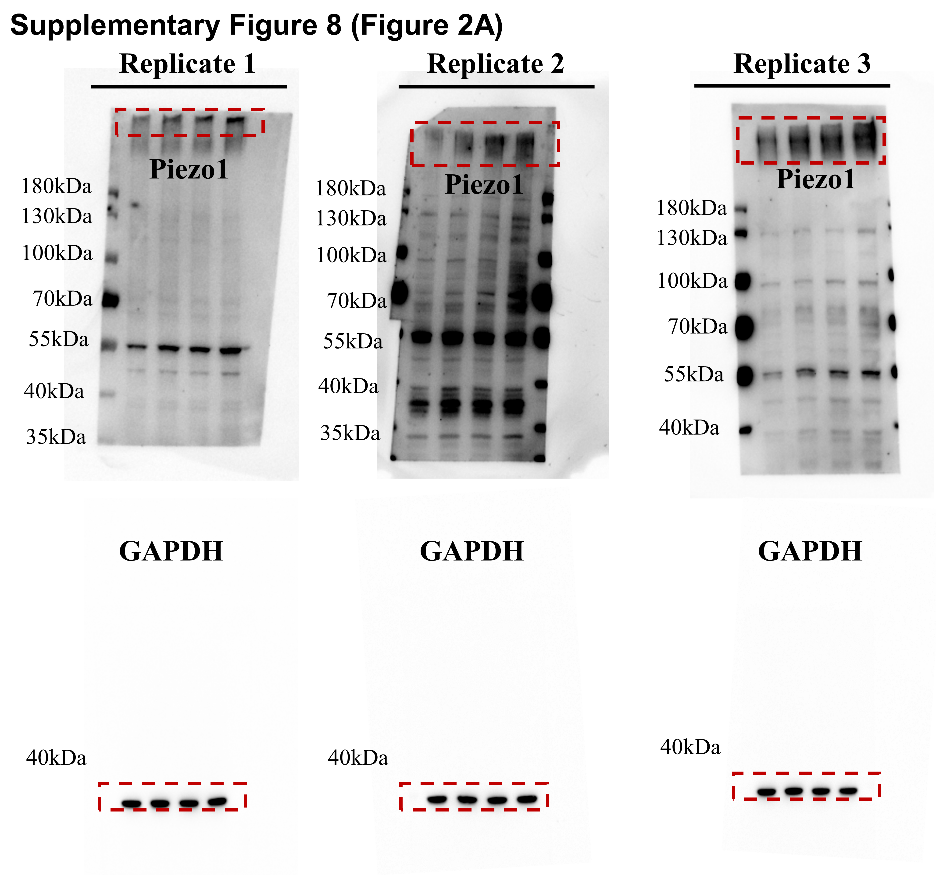


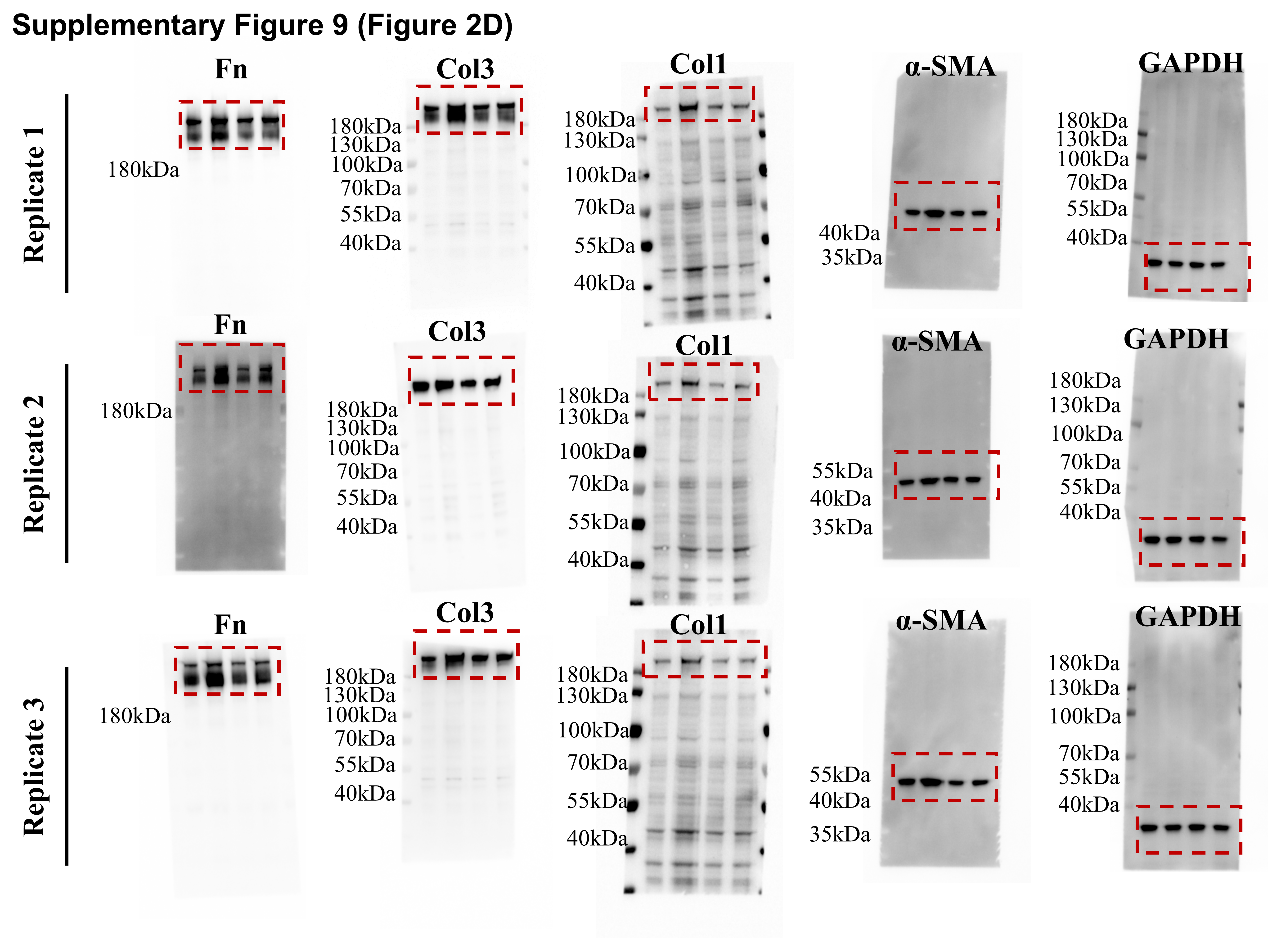


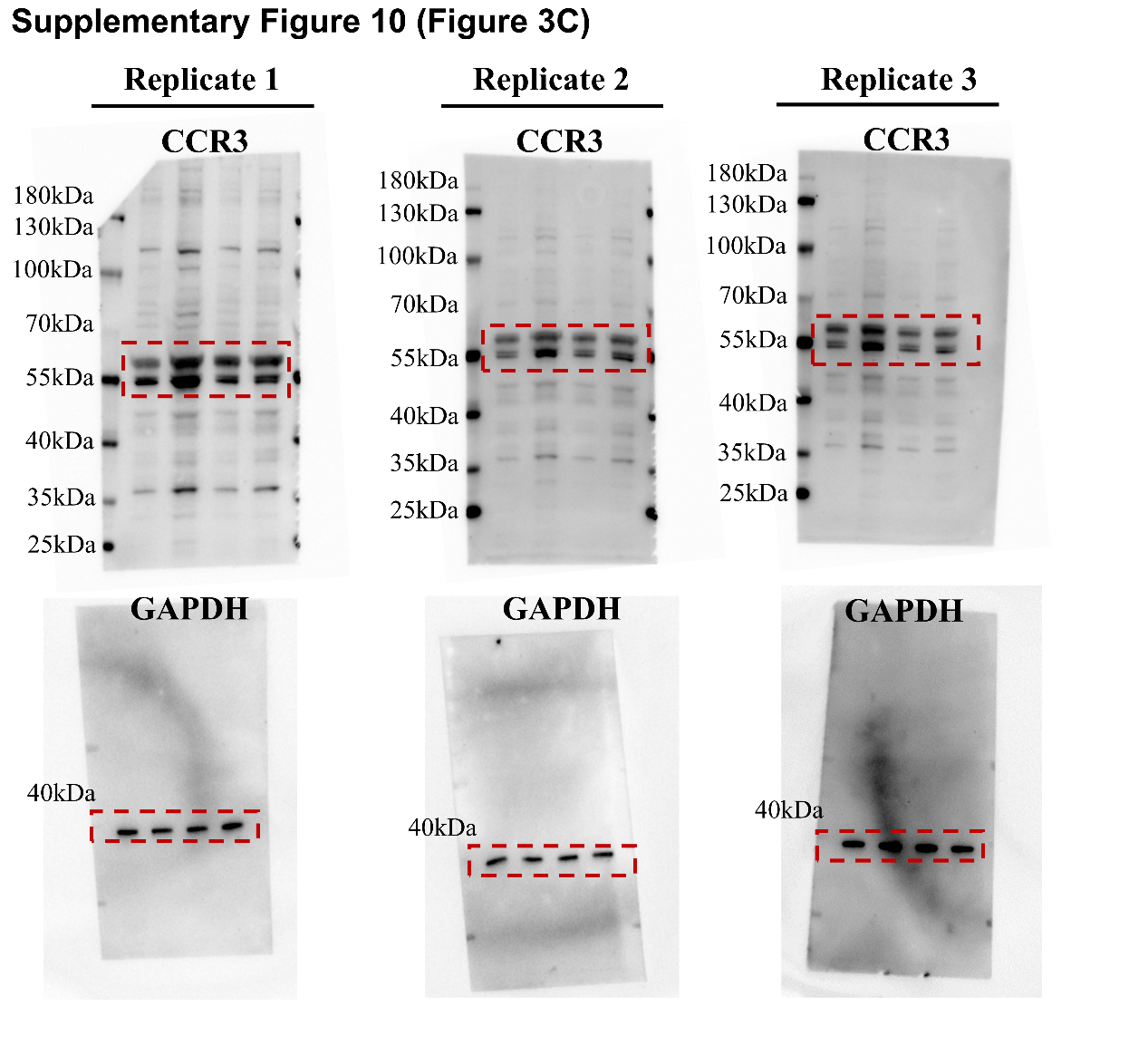


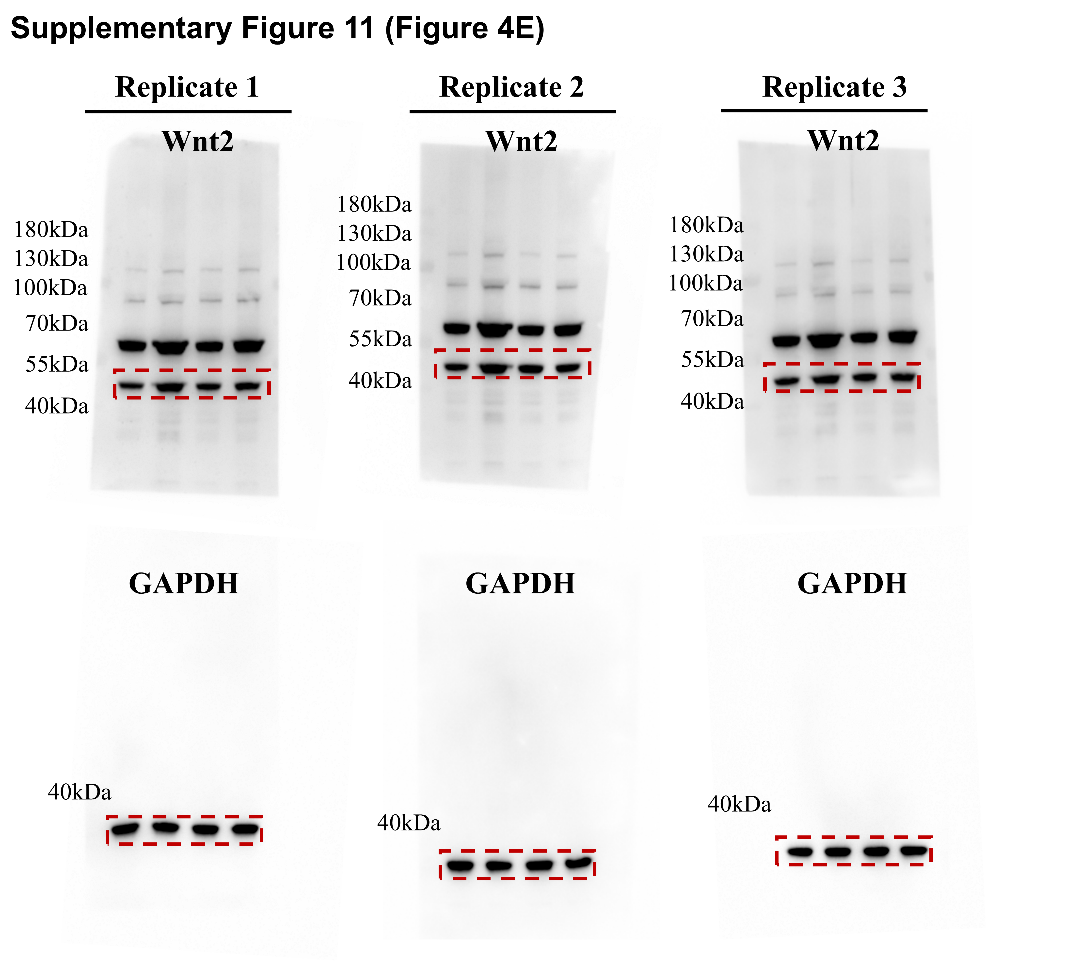


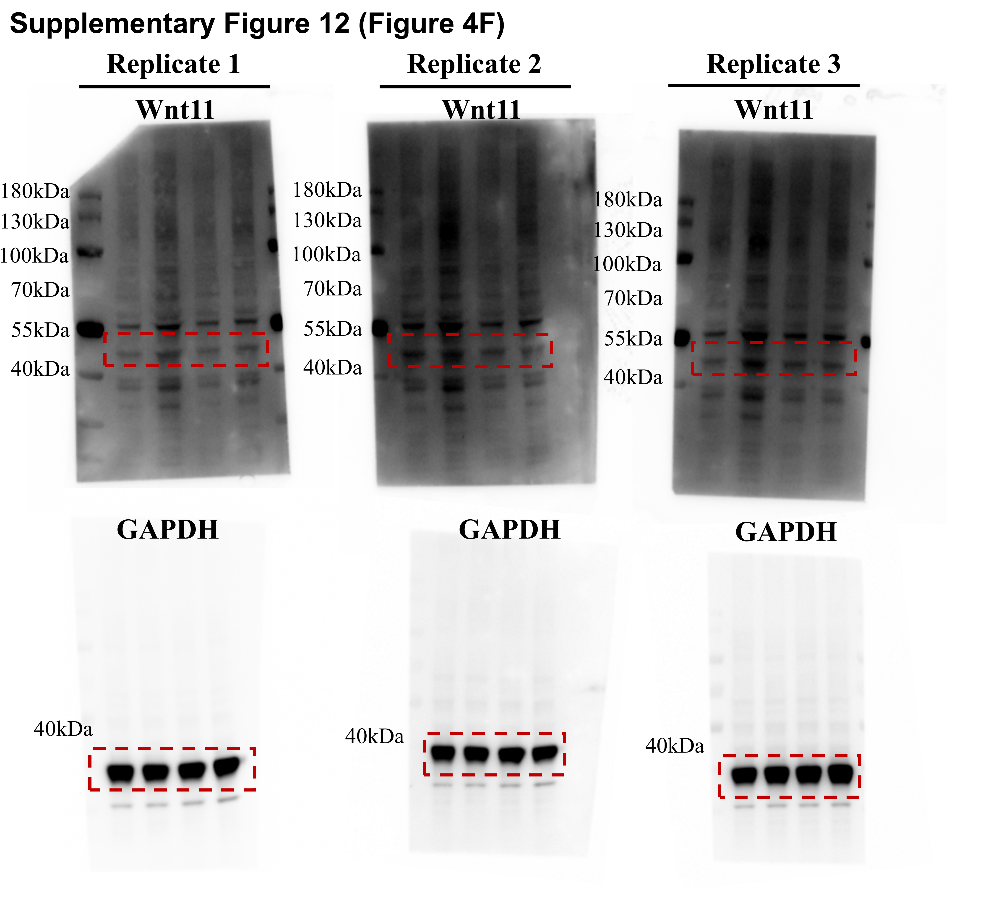


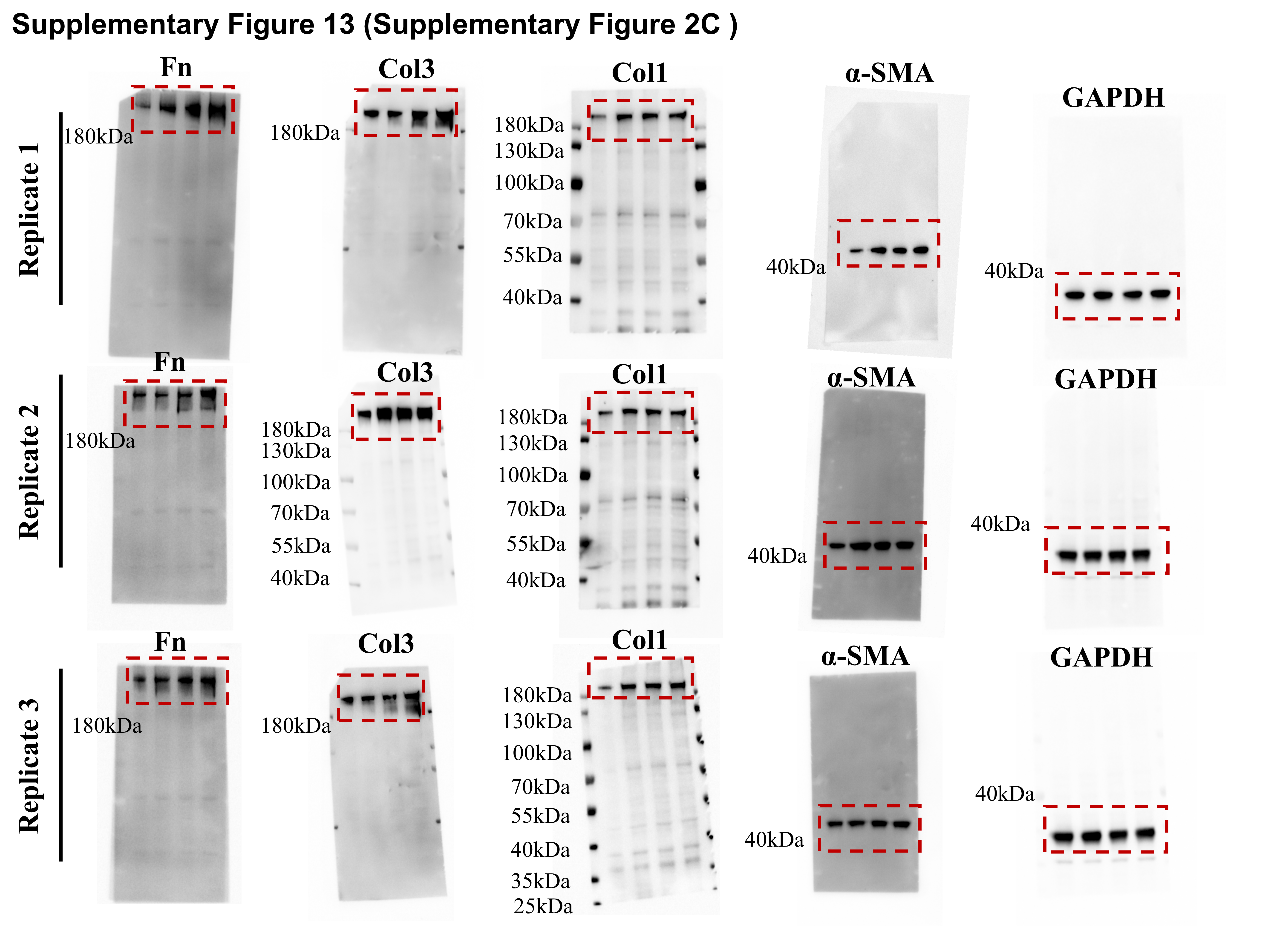


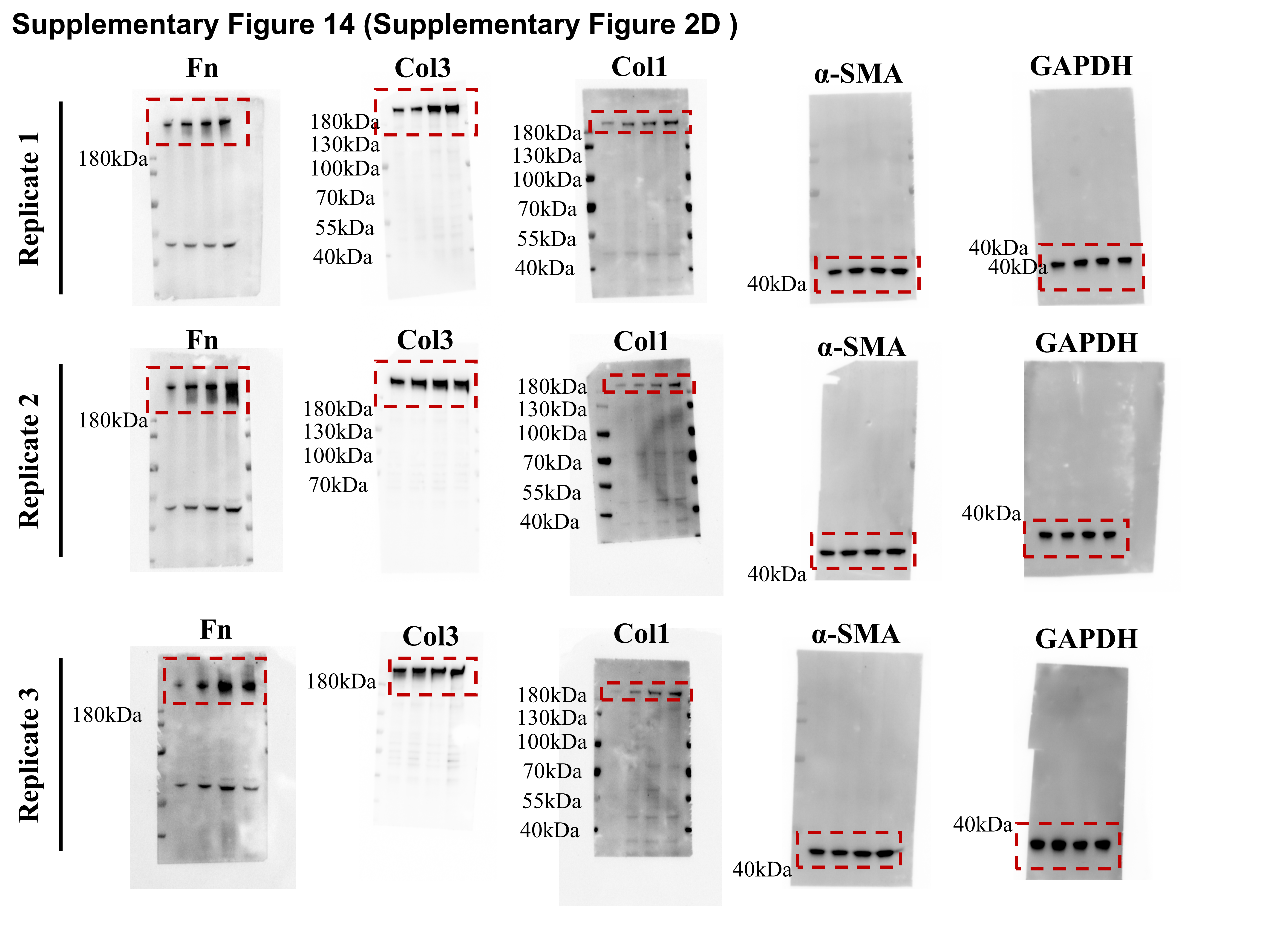


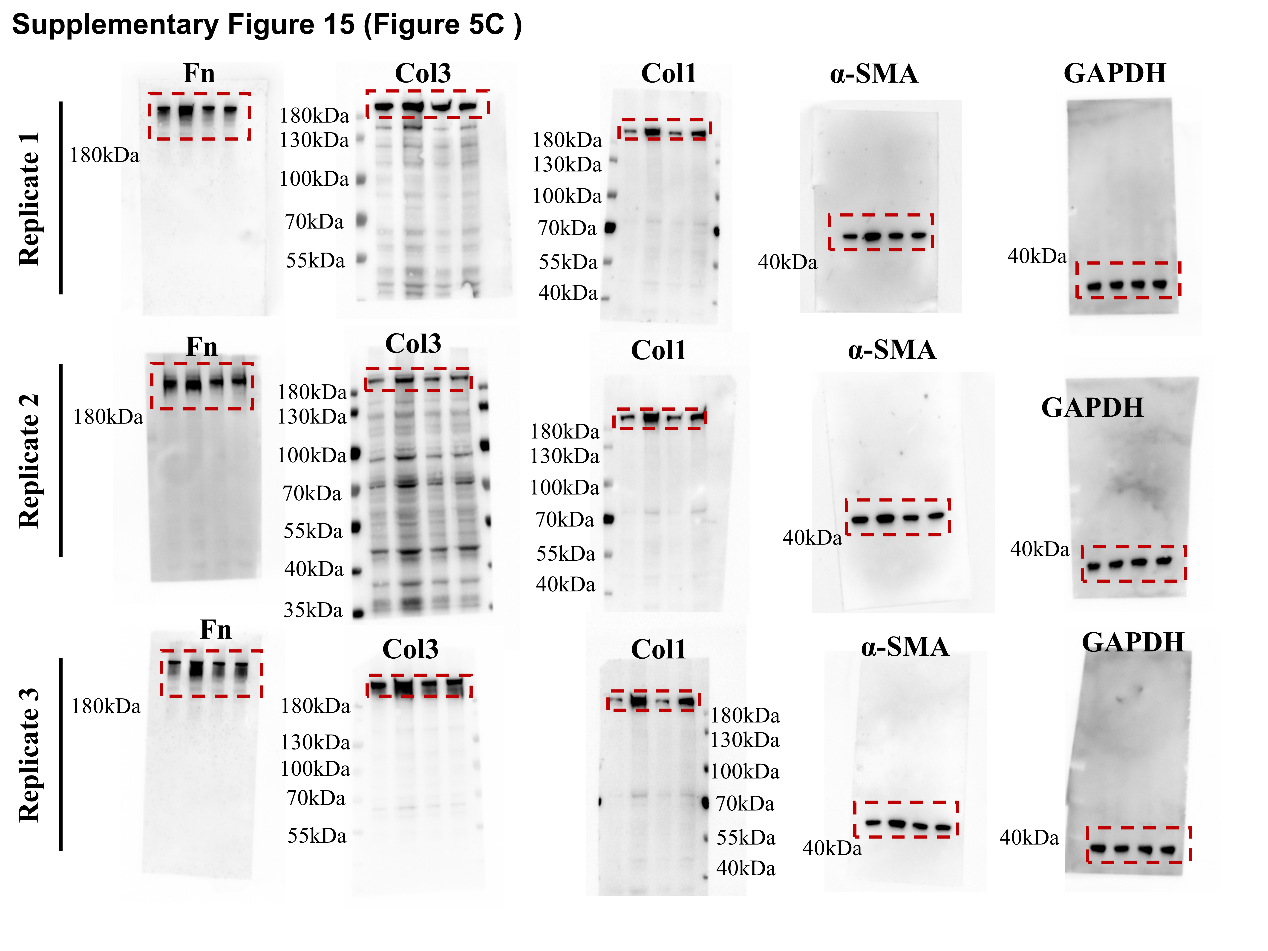


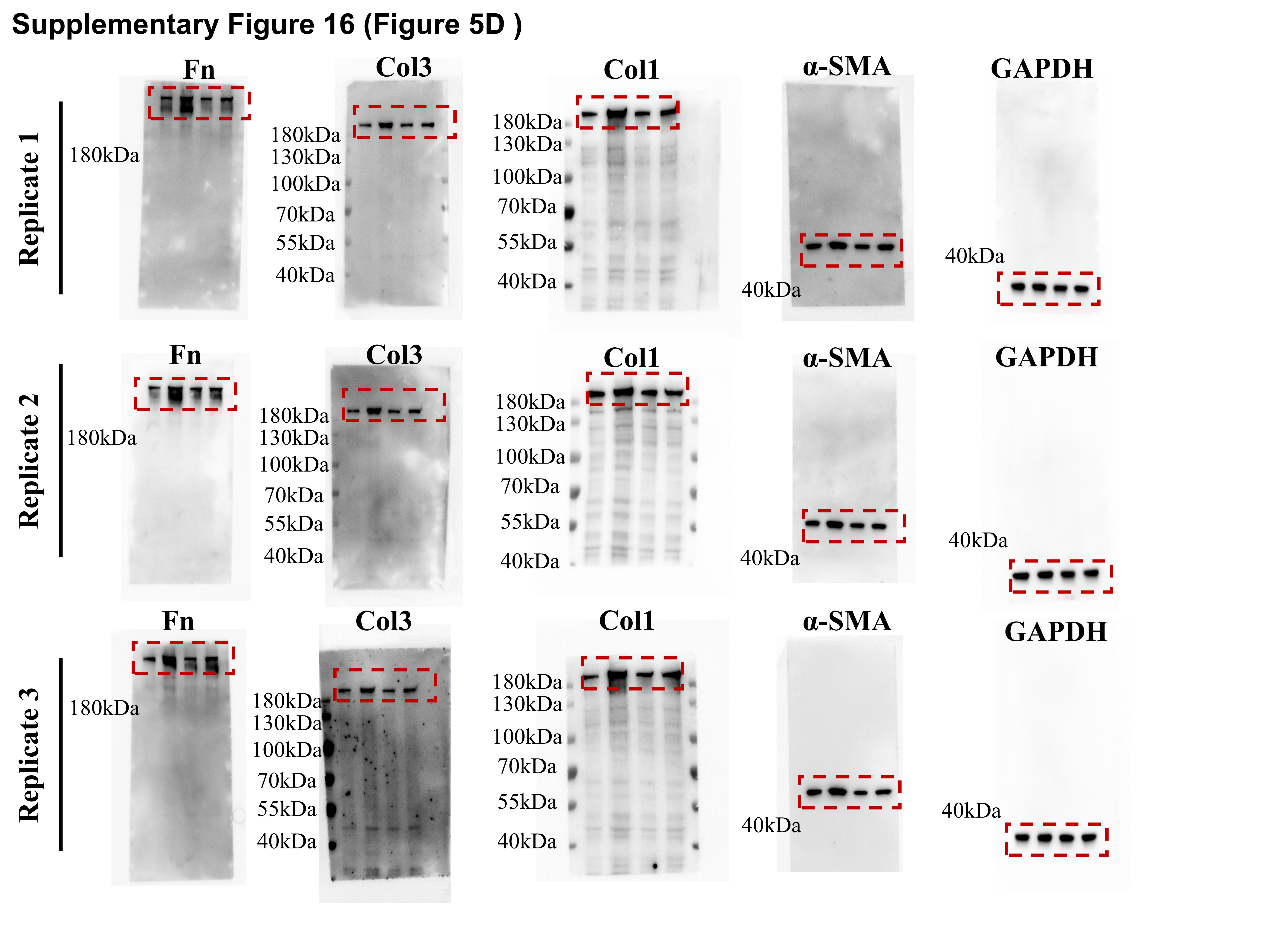


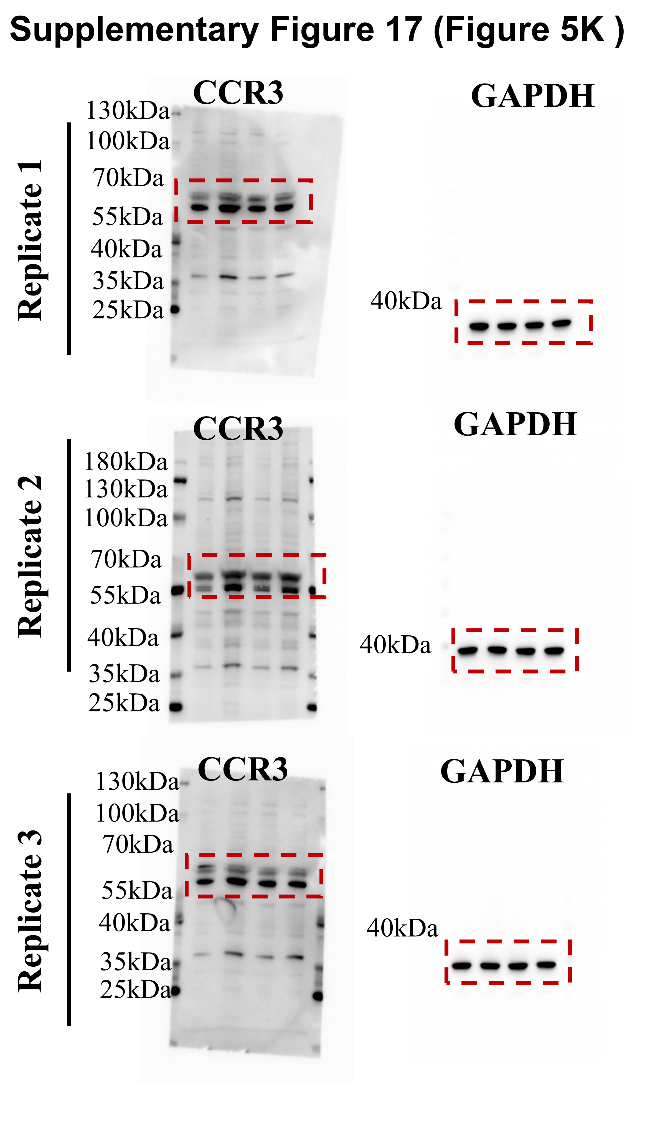

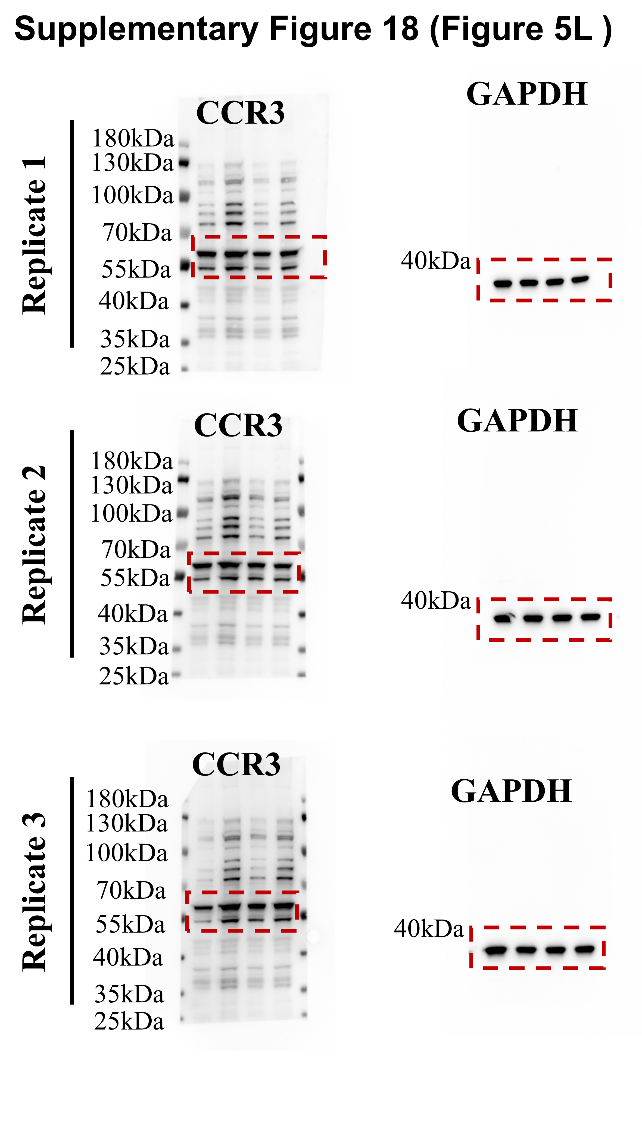


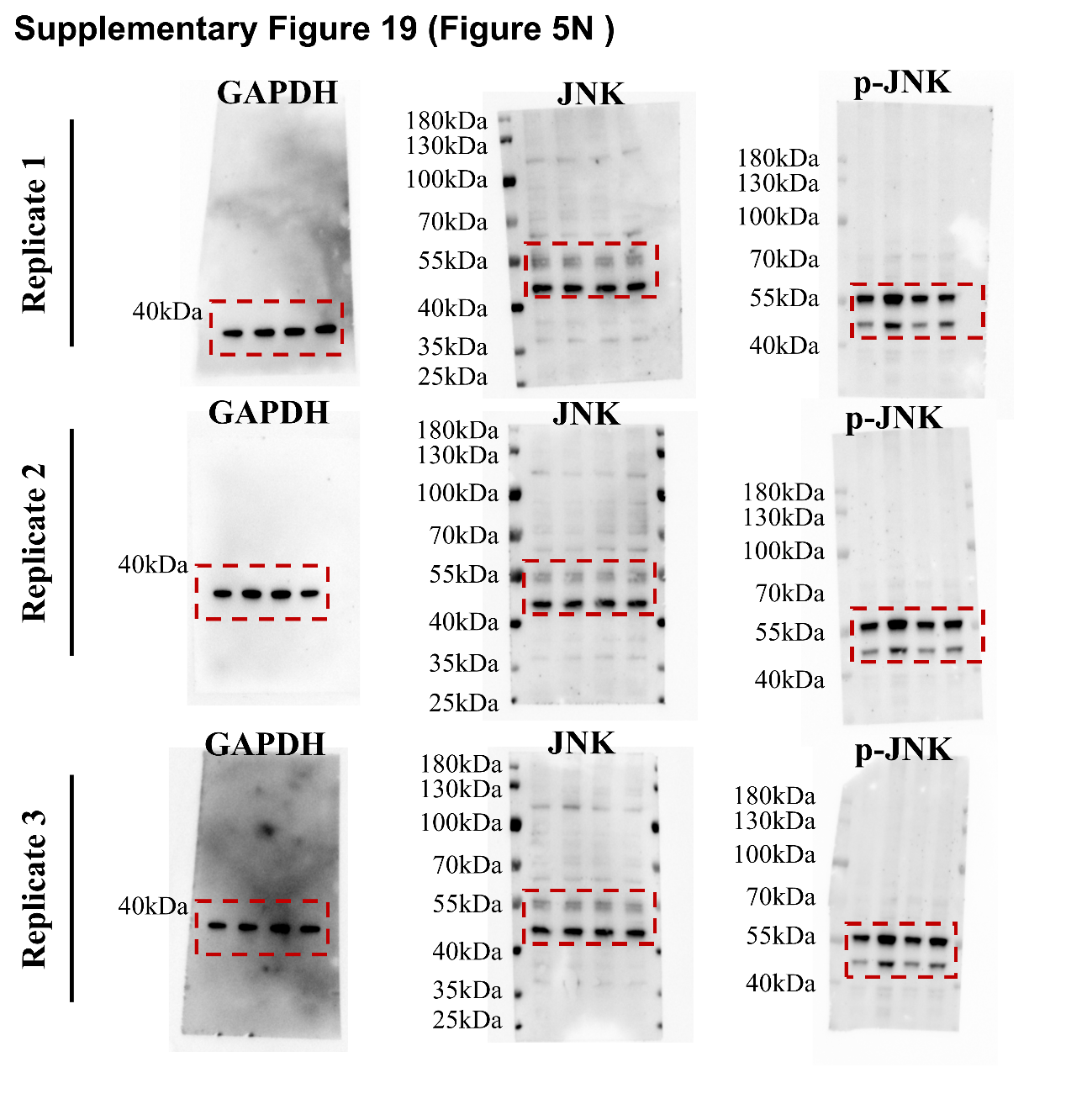


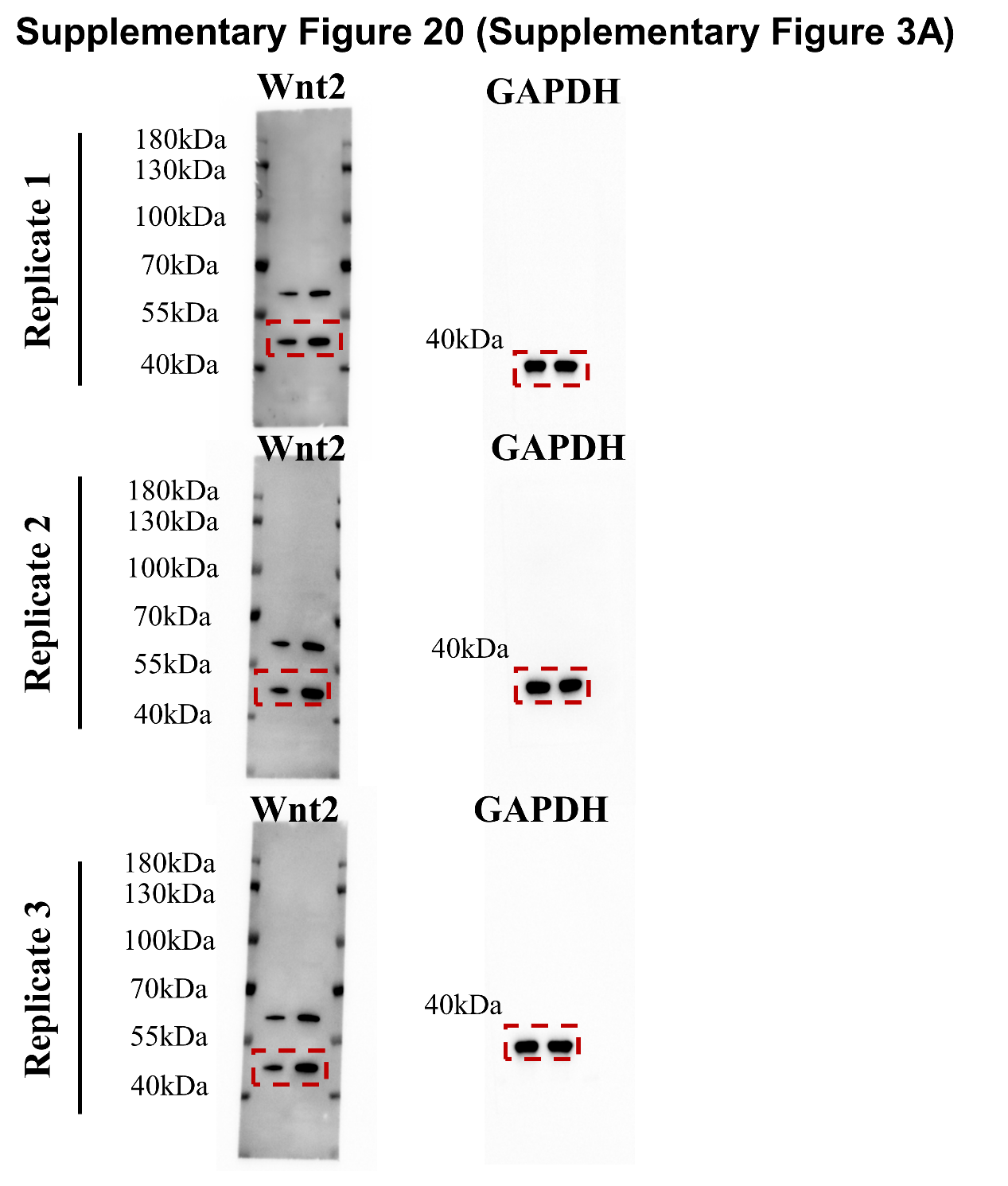


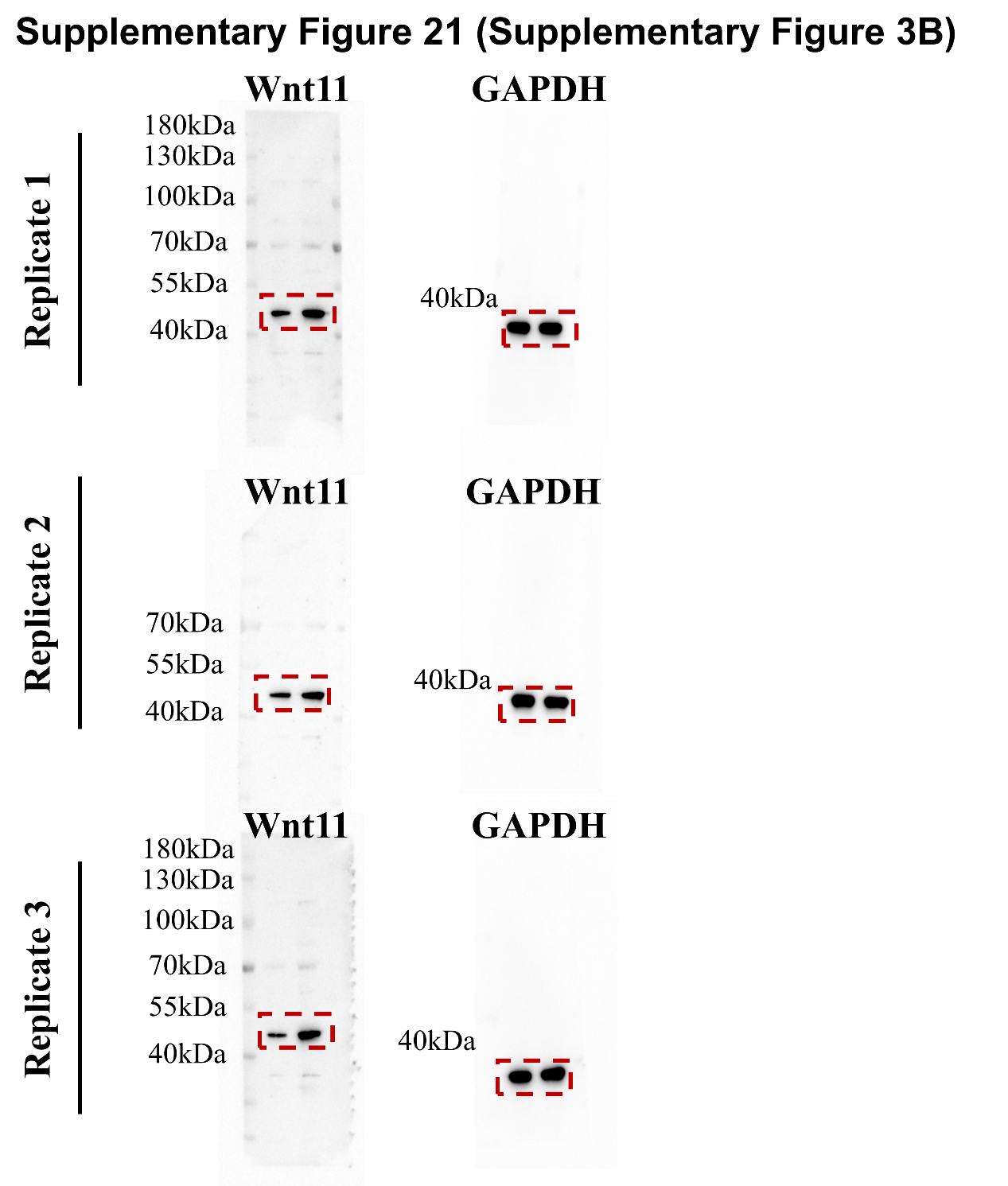


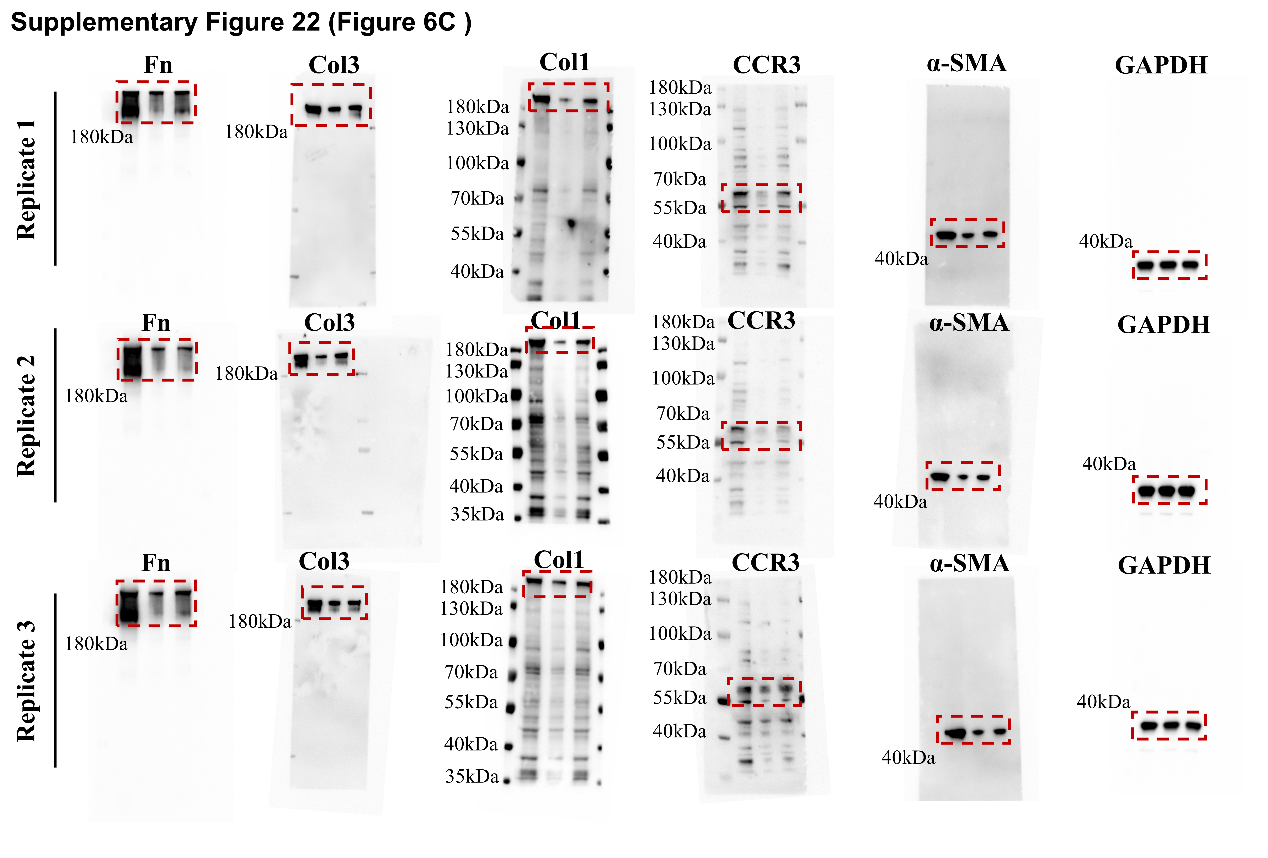


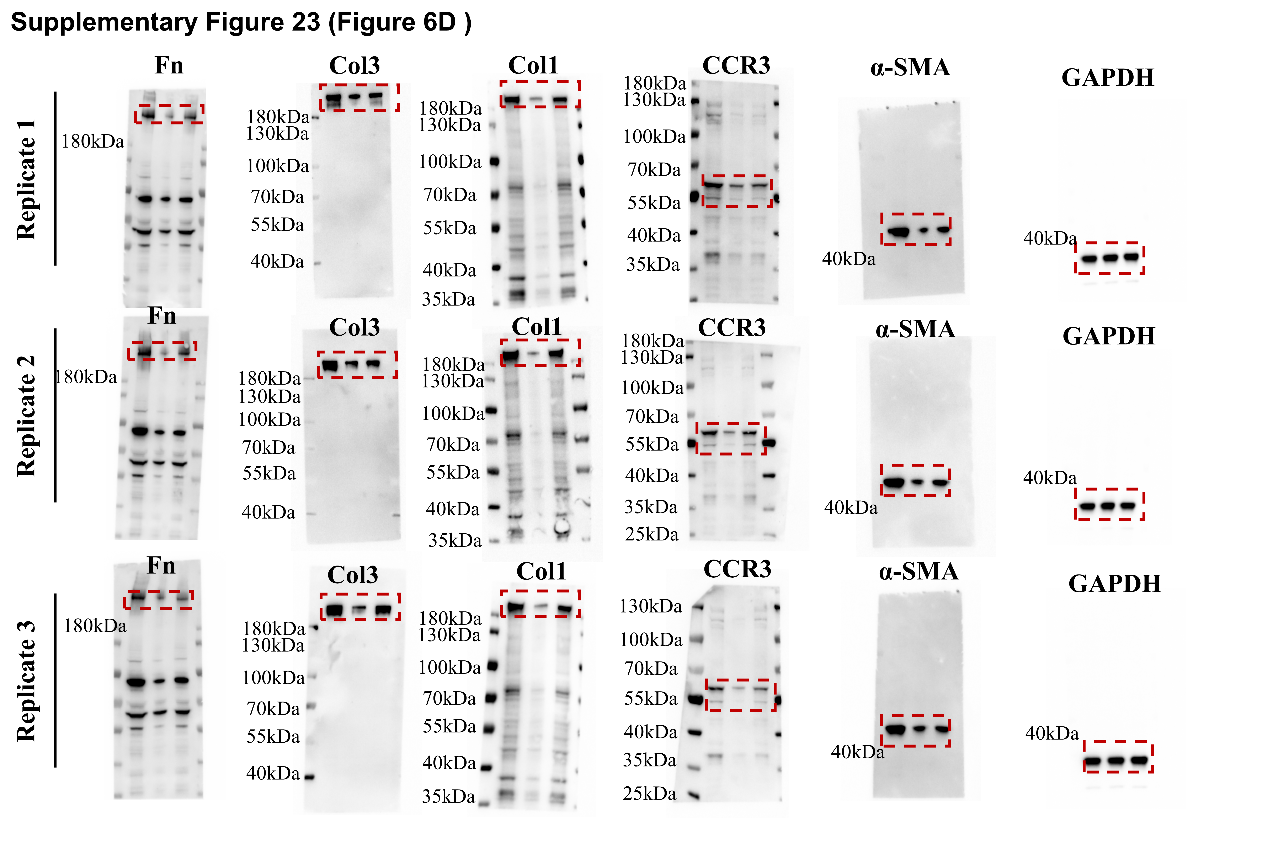

Supplement: Supplementary file 2 — Supplemental materials [file 41419_2024_6466_MOESM2_ESM.docx]
