# Supplementary material for: Mechanical stiffness promotes skin fibrosis via Piezo1-Wnt2/Wnt11-CCL24 positive feedback loop
Source: Cell Death Dis. 2024 Jan 24;15(1):84. doi: 10.1038/s41419-024-06466-3 (PMC10808102; doi:10.1038/s41419-024-06466-3)

Supplementary Figure 6 (Supplementary Figure 1A upper part)

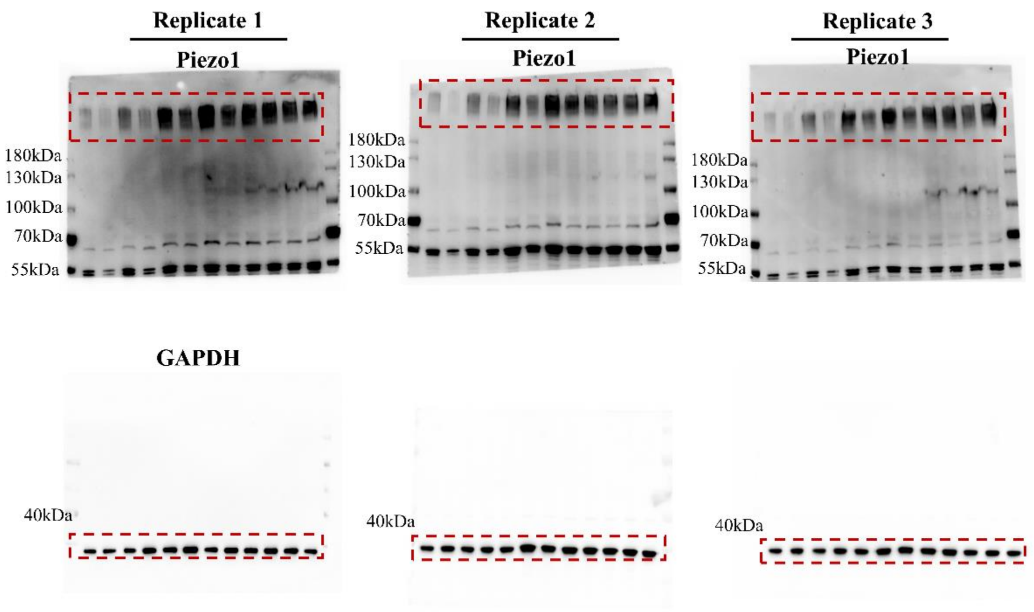

Supplementary Figure 6 (Supplementary Figure 1A bottom part)

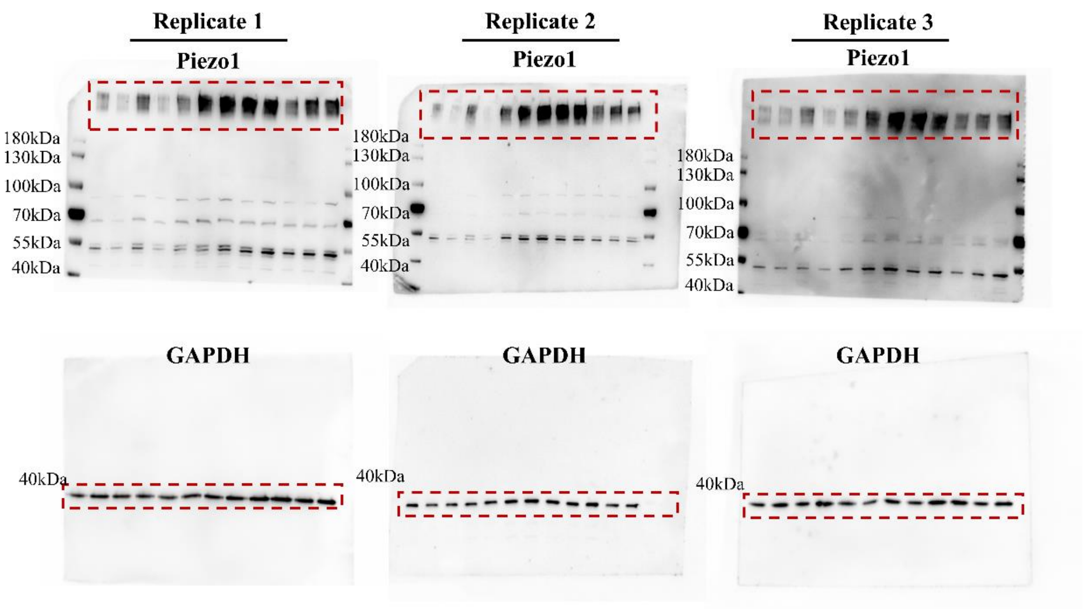

Supplementary Figure 7 (Supplementary Figure 1B)

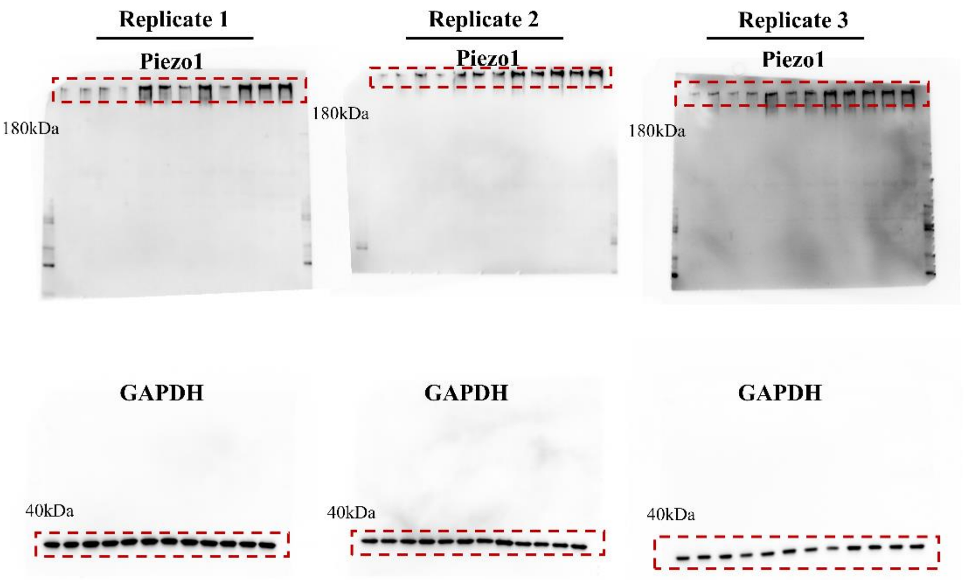

Supplementary Figure 8 (Figure 2A)

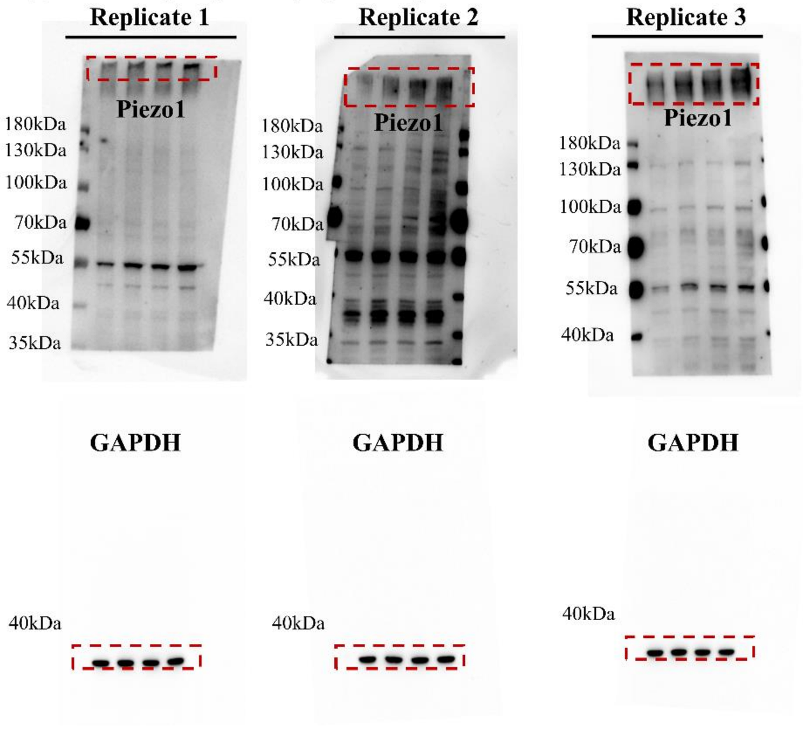

Supplementary Figure 9 (Figure 2D)

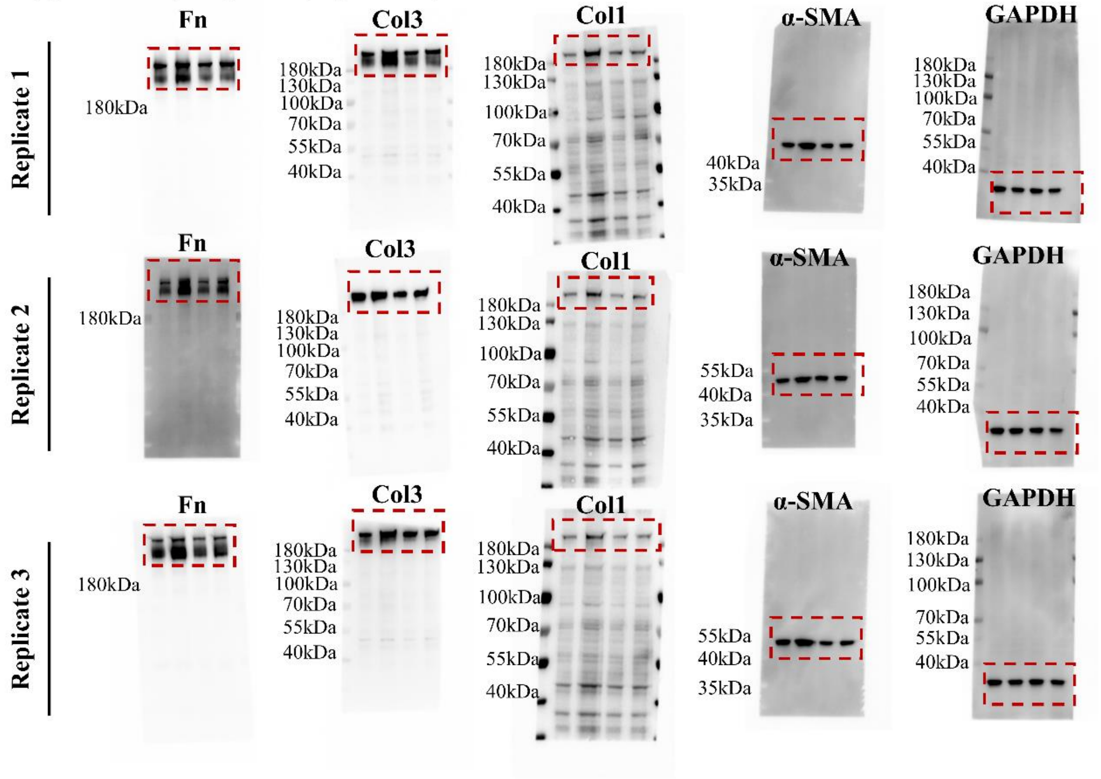

Supplementary Figure 10 (Figure 3C)

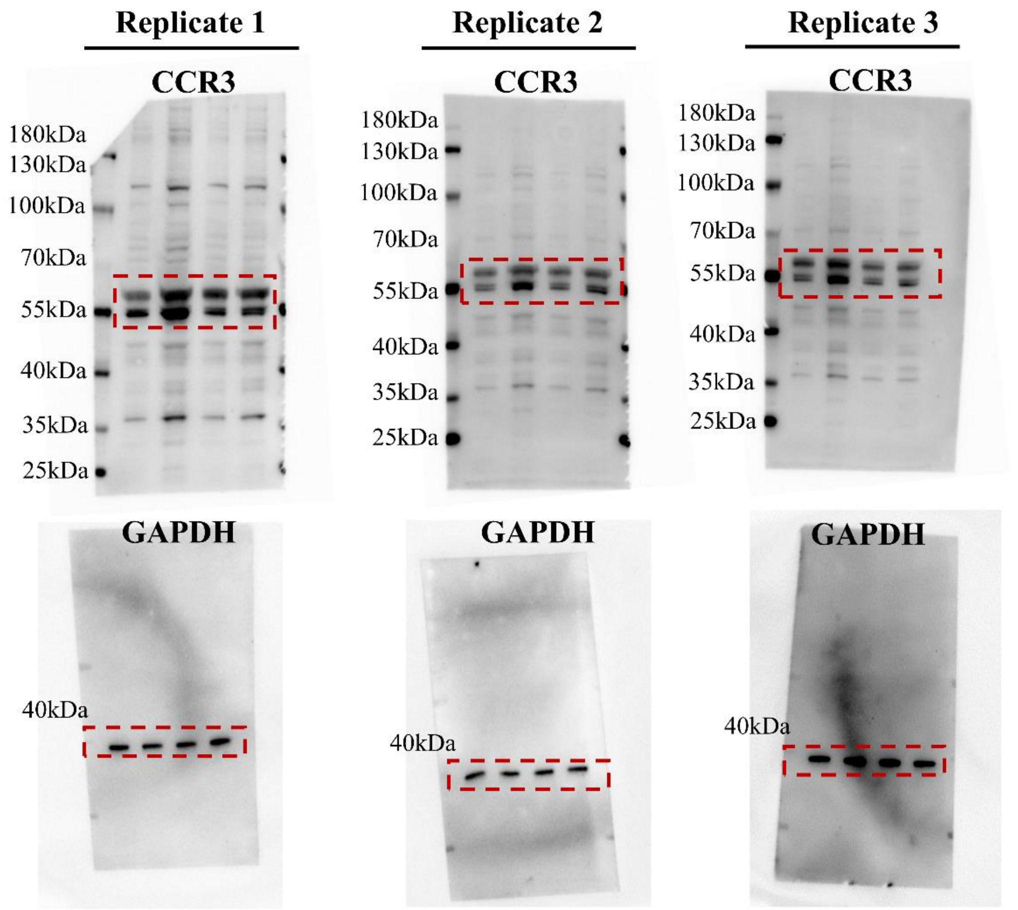

Supplementary Figure 11 (Figure 4E)

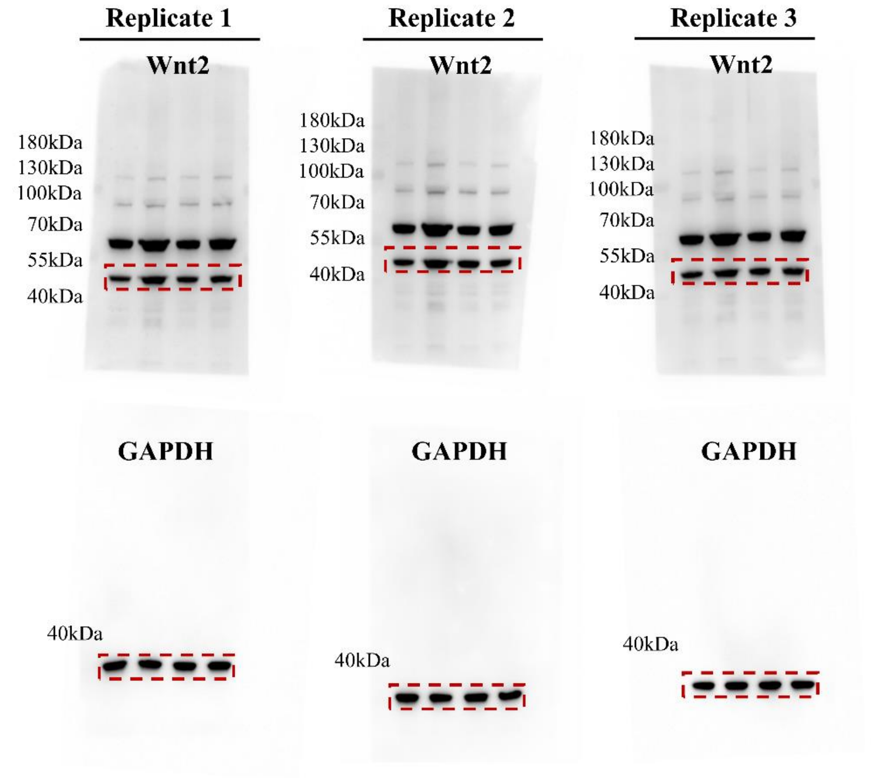

Supplementary Figure 12 (Figure 4F)

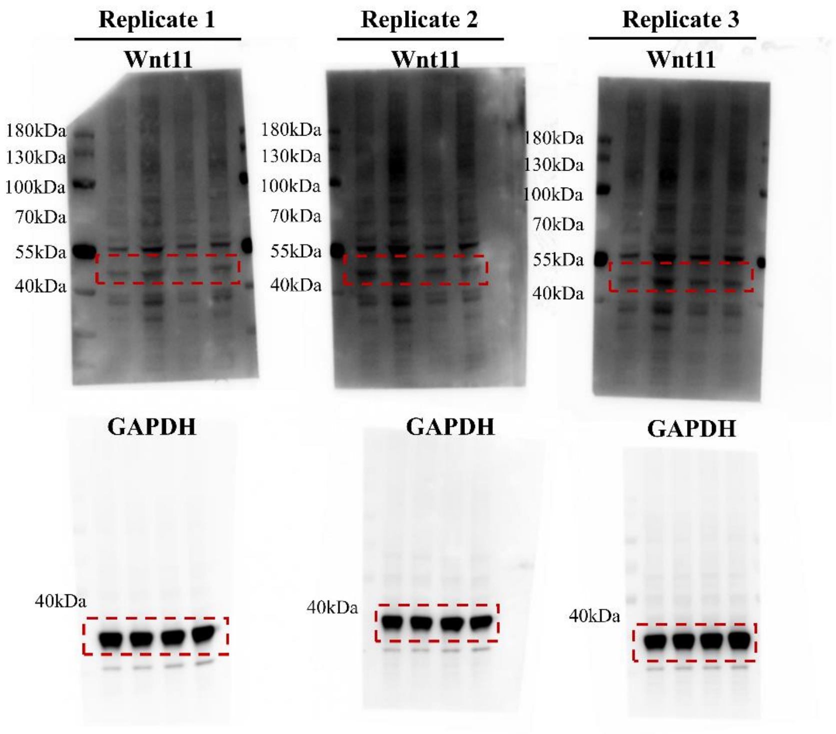

Supplementary Figure 13 (Supplementary Figure 2C)

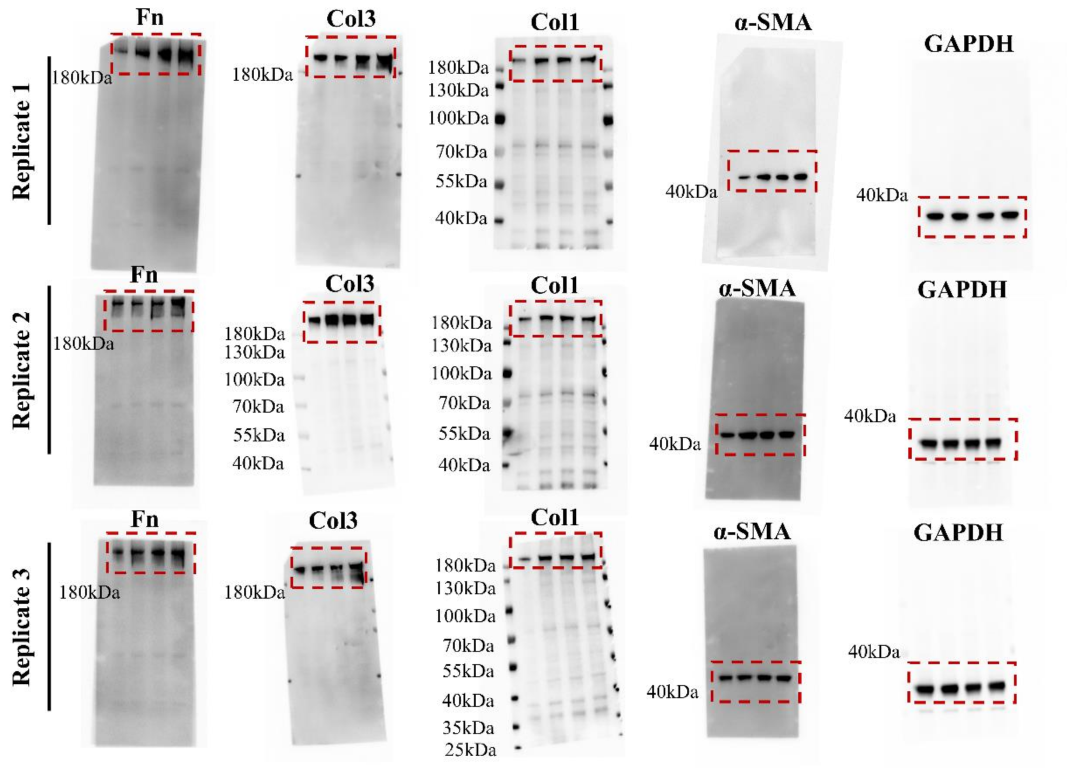

Supplementary Figure 14 (Supplementary Figure 2D)

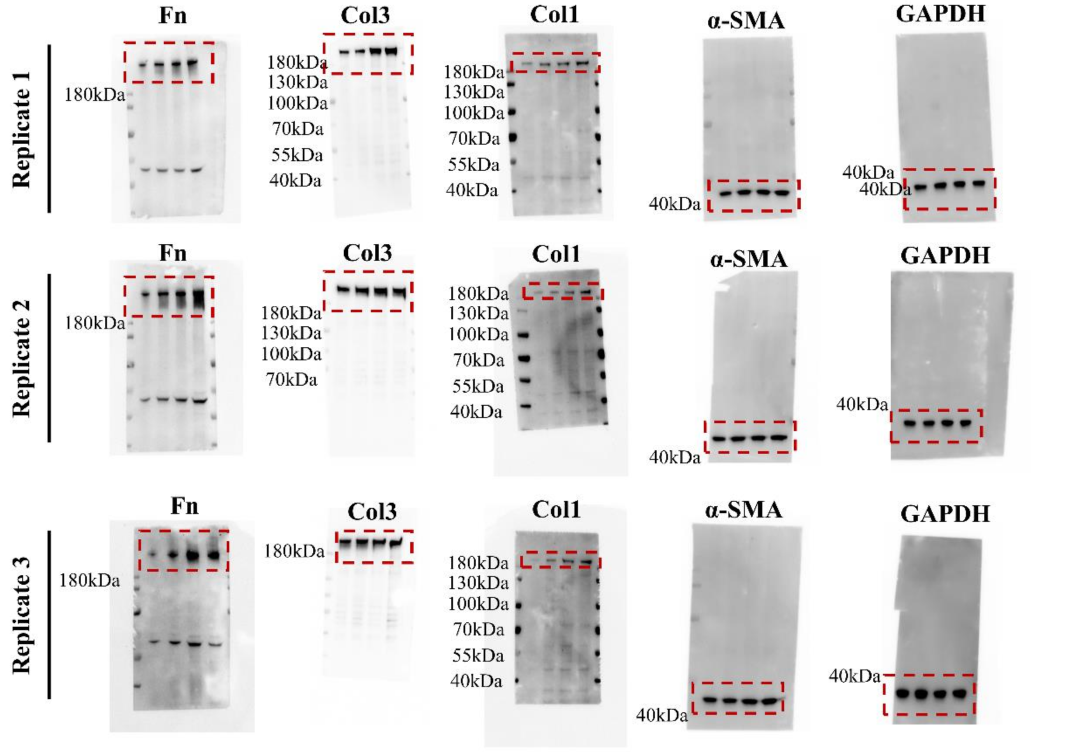

Supplementary Figure 15 (Figure 5C)

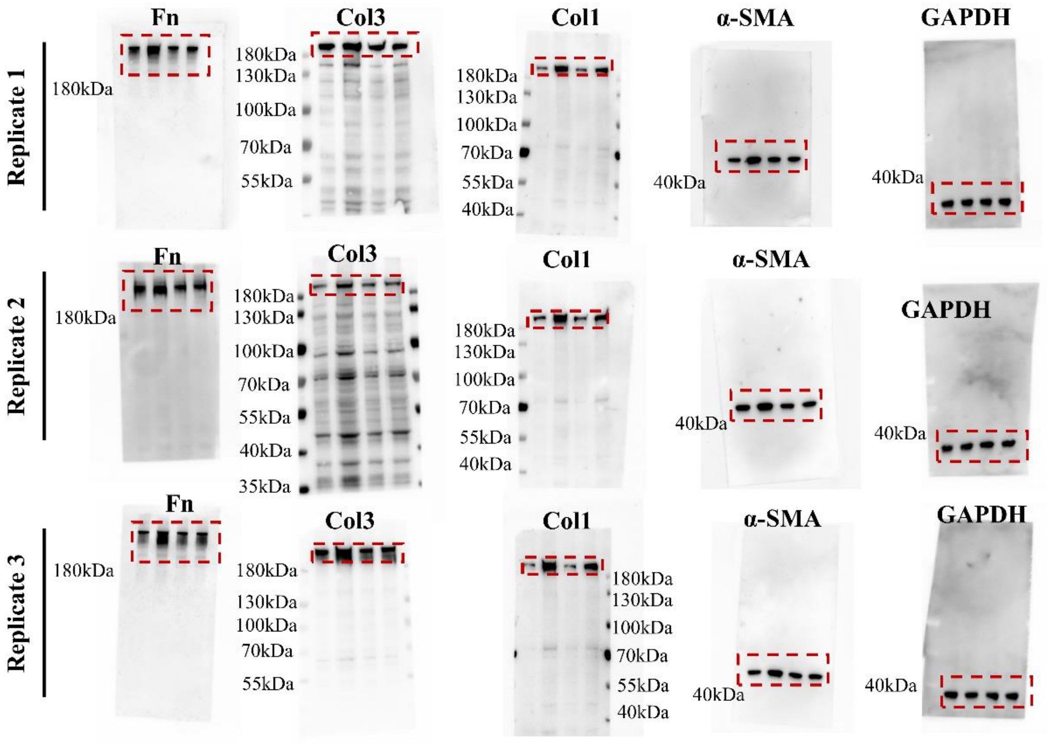

Supplementary Figure 16 (Figure 5D)

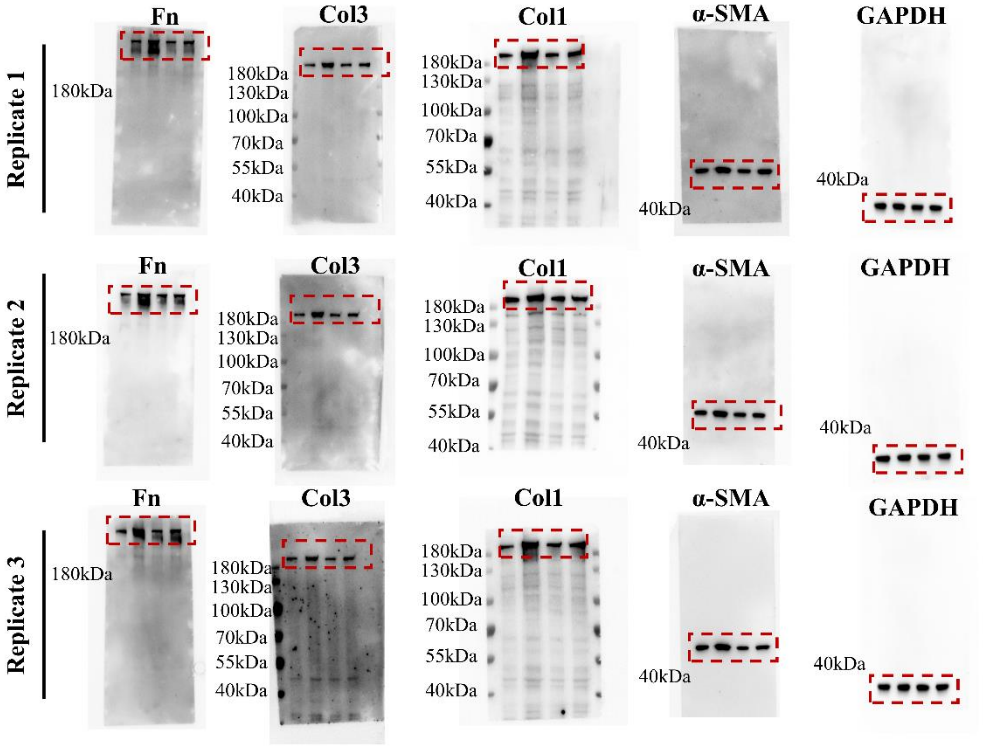

Supplementary Figure 17 (Figure 5K)

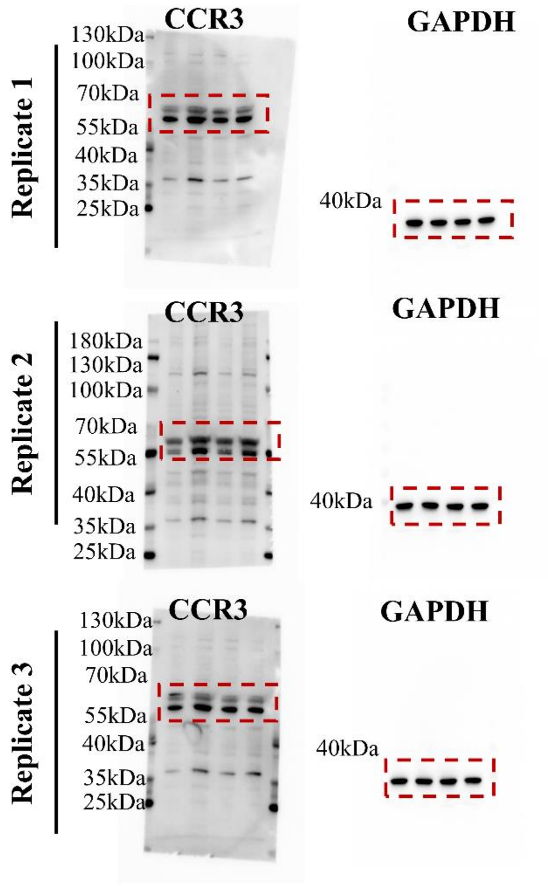

Supplementary Figure 18 (Figure 5L)

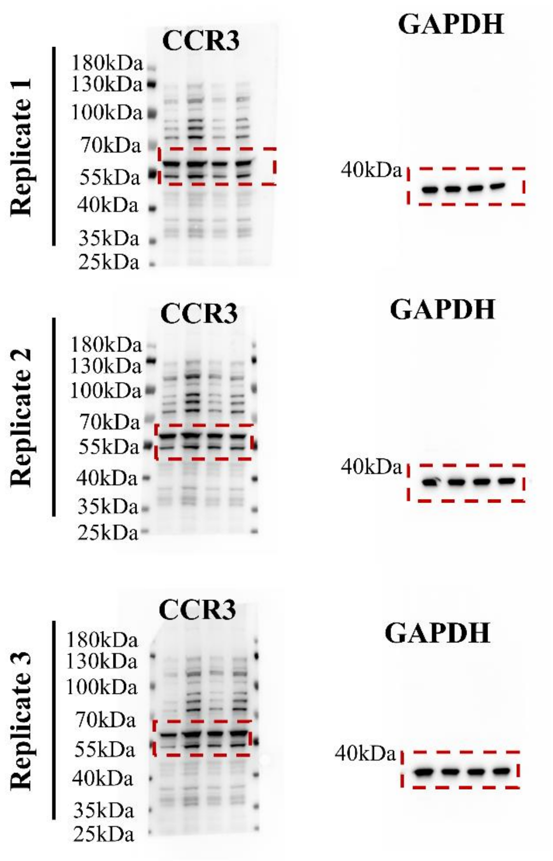

Supplementary Figure 19 (Figure 5N)

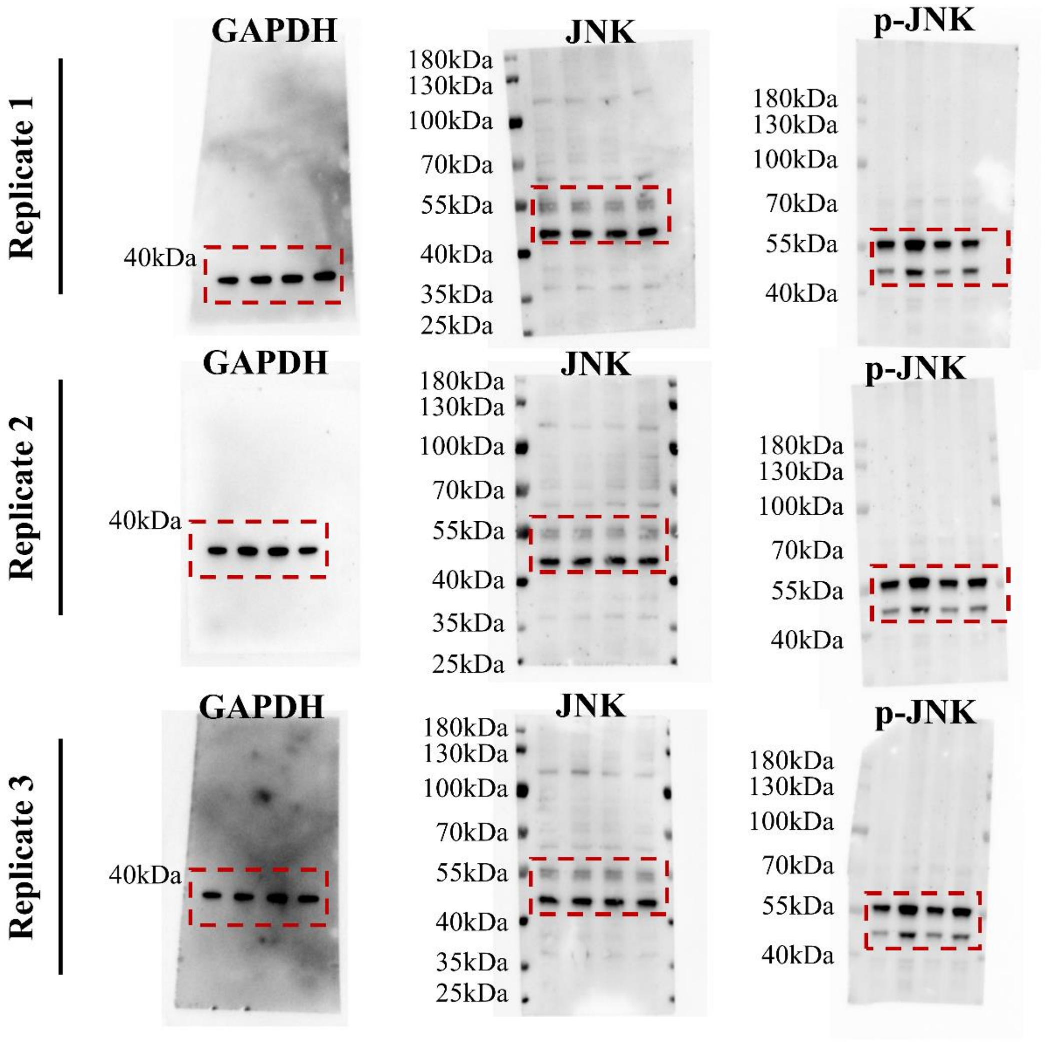

Supplementary Figure 20 (Supplementary Figure 3A)

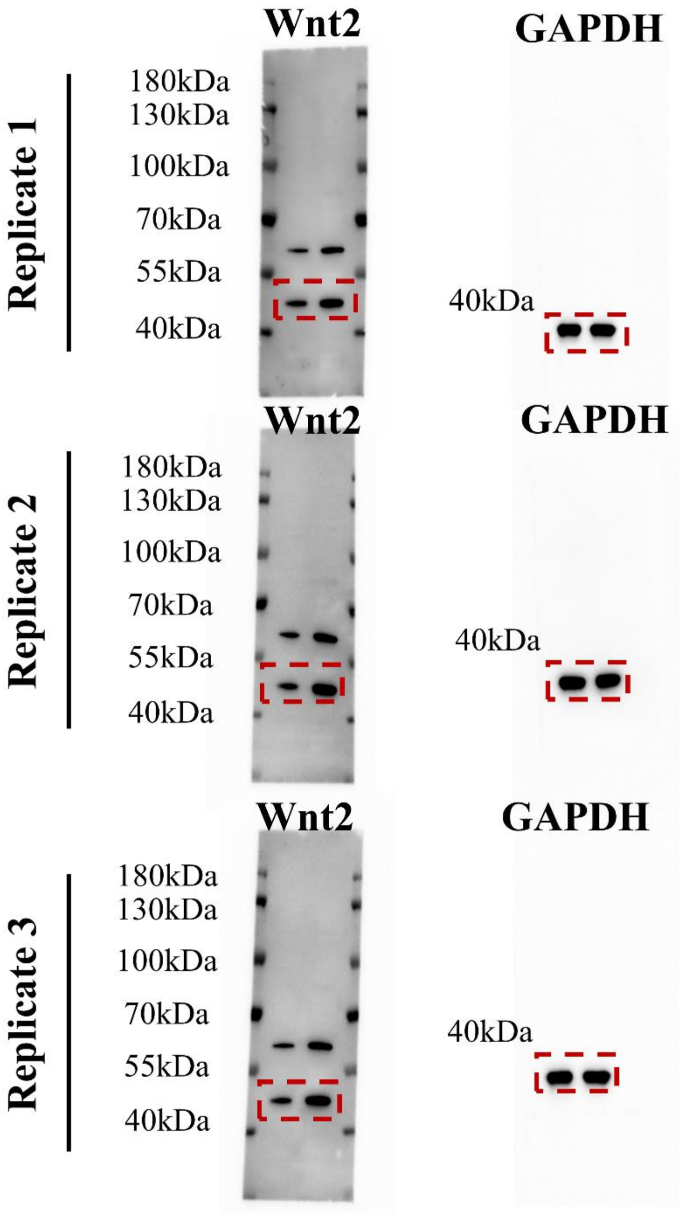

Supplementary Figure 21 (Supplementary Figure 3B)

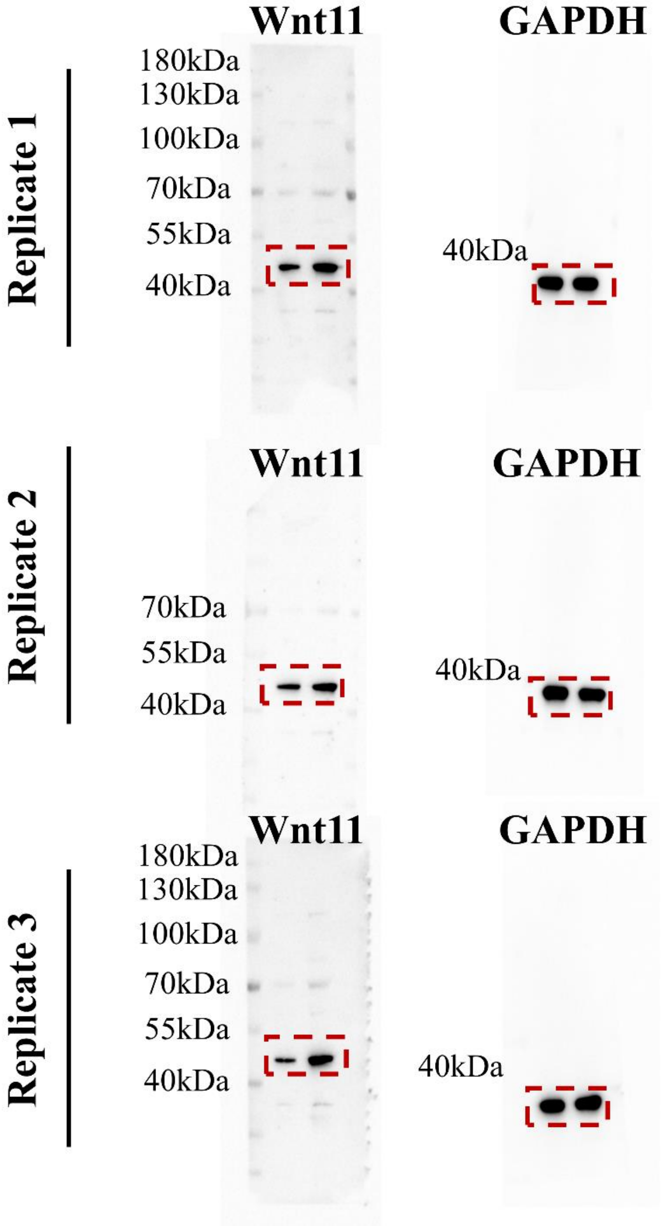

Supplementary Figure 22 (Figure 6C)

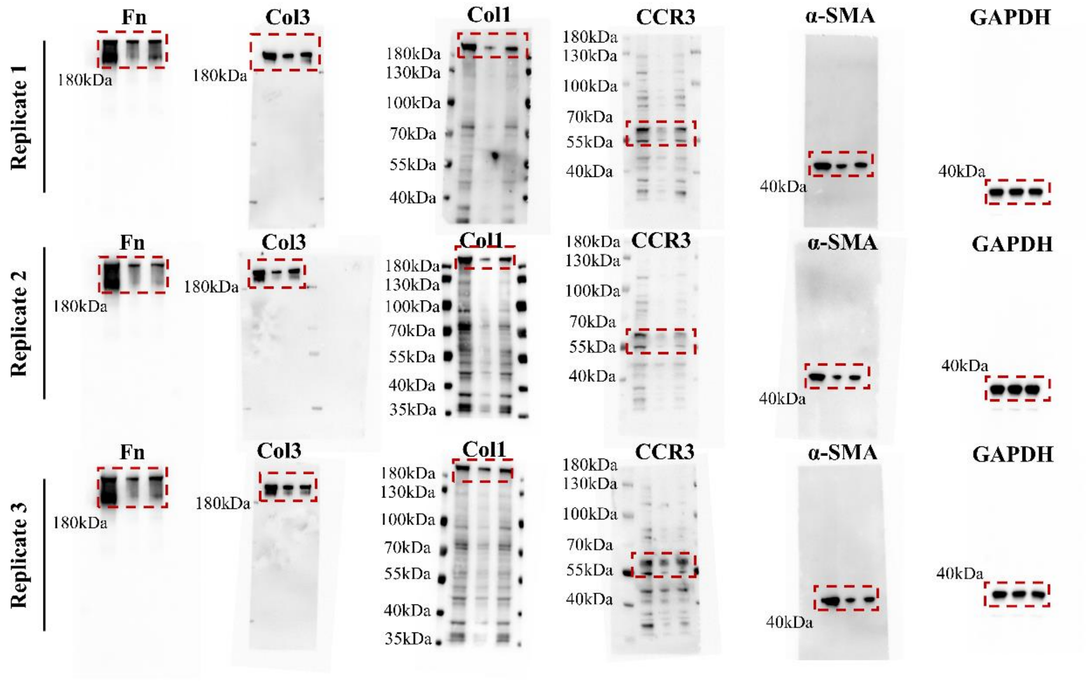

Supplementary Figure 23 (Figure 6D)

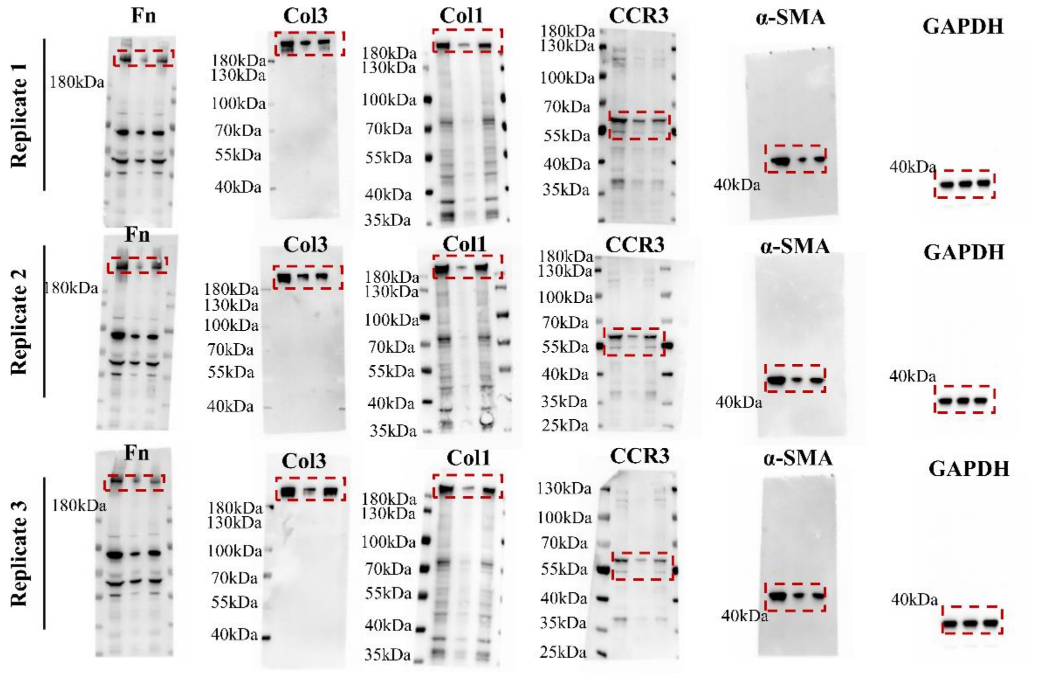

Supplement: Supplementary file 3 — Full-length western blots [file 41419_2024_6466_MOESM3_ESM.pdf]
